# Whole Genome Sequencing Provides Information on the Genomic Architecture and Diversity of Cultivated Gilthead Seabream (*Sparus aurata*) Broodstock Nuclei

**DOI:** 10.3390/genes14040839

**Published:** 2023-03-30

**Authors:** Francesca Bertolini, Anisa Ribani, Fabrizio Capoccioni, Luca Buttazzoni, Samuele Bovo, Giuseppina Schiavo, Massimo Caggiano, Max F. Rothschild, Luca Fontanesi

**Affiliations:** 1 Department of Agricultural and Food Sciences, Division of Animal Sciences, University of Bologna, Viale G. Fanin 46, 40127 Bologna, Italy; 2 Centro di Ricerca “Zootecnia e Acquacoltura”, Consiglio per la Ricerca in Agricoltura e l’Analisi dell’Economia Agraria (CREA), 00198 Roma, Italy; 3 Panittica Italia Società Agricola Srl, Torre Canne di Fasano, 72016 Brindisi, Italy; 4 Department of Animal Science, Iowa State University, Ames, IA 50011-3150, USA

**Keywords:** aquaculture, DNA pool-seq, FST, population genetics, signatures of selection, SNP

## Abstract

The gilthead seabream (*Sparus aurata*) is a species of relevance for the Mediterranean aquaculture industry. Despite the advancement of genetic tools for the species, breeding programs still do not often include genomics. In this study, we designed a genomic strategy to identify signatures of selection and genomic regions of high differentiation among populations of farmed fish stocks. A comparative DNA pooling sequencing approach was applied to identify signatures of selection in gilthead seabream from the same hatchery and from different nuclei that had not been subjected to genetic selection. Identified genomic regions were further investigated to detect SNPs with predicted high impact. The analyses underlined major genomic differences in the proportion of fixed alleles among the investigated nuclei. Some of these differences highlighted genomic regions, including genes involved in general metabolism and development already detected in QTL for growth, size, skeletal deformity, and adaptation to variation of oxygen levels in other teleosts. The obtained results pointed out the need to control the genetic effect of breeding programs in this species to avoid the reduction of genetic variability within populations and the increase in inbreeding level that, in turn, might lead to an increased frequency of alleles with deleterious effects.

## 1. Introduction

Gilthead seabream (*S. aurata*) is one of the most relevant species of the Mediterranean aquaculture industry, with an annual production that exceeds 208,808 metric tons, mainly obtained in Turkey, Greece, Croatia, and Italy [1]. The development in seabream aquaculture has been associated with the robustness and plasticity of this species, highly adaptable to different environments and resilient to diet changes and microbial outbreaks [2]. Technological advances in reproduction, larval rearing conditions, production of high-quality feed, disease prevention, and environmental control have supported the expansion of this industry [2,3].

To further support these developments, breeding programs have recently been initiated in Europe to reduce deformities while improving growth, flesh quality, feed efficiency, reproduction, and disease resistance in cultivated gilthead seabream populations, mainly starting from wild and heterogeneous stocks [2,4,5]. In this context, genetic characterization of these semi-domesticated populations might be useful to see how breeding programmes are shaping their genetic background and to evaluate their genetic potential.

More recent advancements at the genomic level, including the production of a complete reference genome for the species [6] and the development of a Single Nucleotide Polymorphism (SNP) array for high throughput genotyping of more than 30,000 SNPs [7], have provided important tools and information to explore cultivated genetic resources of gilthead seabream. These tools have allowed some first attempts to dissect the genetic architecture of economically relevant traits in *S. aurata*, such as feed efficiency [8], disease resistance [9,10,11], deformity [12], and pigmentation defects [13].

Genomic information can also be useful to investigate the effects of both natural and artificial selection in fish species and to identify signatures of selection that are the footprints left across the genome [14]. These regions might usually harbour functionally important variants that could provide a selective advantage due to their potential effects on economically relevant traits [14,15,16].

Thus far, several population genetic studies have been carried out in gilthead seabream mainly investigating wild and, in few cases, farmed gilthead seabream stocks using mitochondrial DNA markers, microsatellites, or high-density SNP data [7,17,18,19,20,21,22,23,24,25]. Population genetic structures caused by geographic distances, genetic drift, and bottlenecks could be captured. Few of these studies have also attempted to identify signatures of selection in the genome of this species using different types of DNA markers [24,25]. As far as we know, whole genome sequencing data have not been extensively analysed thus far in *S. aurata* for the same purposes. Whole-genome sequencing of DNA pools constituted from many individuals (Pool-seq) is a cost-effective approach to determine unbiased genome-wide allele frequencies in populations under investigation [26].

In this study, we applied a comparative DNA pooling sequencing approach in gilthead seabream from farmed genetic stocks to detect signatures of selection in the genome of this species. The experimental design was based on the genomic analysis of offspring of different broodstock nuclei out of the same hatchery. As gilthead seabream are mass spawners, reproduction in hatchery facilities is managed in broodstock nuclei that contain several males and females to generate offspring that can be tracked back to the nucleus from which they were generated. These nuclei are not subjected to genetic base selection and were compared to detect genomic regions of high divergence possibly originated by some genetic advantage or by genetic drift and bottleneck effects. These regions were further investigated to detect SNPs with high predicted impact. This study designed a genomic strategy to identify signatures of selection and genomic regions of high differentiation among populations of farmed fish stocks and provided a first picture of the impact of gilthead seabream breeding at the genome level.

## 2. Materials and Methods

### 2.1. Gilthead Seabream Populations and Genomic Datasets

The analysed gilthead seabream juveniles were sampled from one commercial hatchery, located in Italy. The samples were offspring from three different broodstock nuclei (nucleus A, B, and C). Each nucleus was composed of 60–87 males and 60–185 females of Atlantic origin. Individual DNA extraction (Promega tissue DNA extraction kit; Promega Corporation, Madison, WI, USA) was carried out from the offspring of these families, which aged approximately 60–66 days post-hatching. Then, five DNA pools were constructed with equimolar contributions of 20 to 30 individual DNA samples: three pools for nucleus A (A1, A2, and A3), one for nucleus B, and one for nucleus C. Whole-genome re-sequencing was carried out from the five DNA pools using Illumina HiSeq 2500, 150 bp paired-end sequencing technology, following the manufacturer’s protocols (Table 1). In addition, raw reads data obtained from 24 DNA pools of wild and farmed seabream data produced by Peñaloza et al. 2021 [7] were retrieved from the Sequence Nucleotide Archive (SRA: PRJEB40423) and used in the first part of the comparative genomic analyses. These raw reads were obtained from farmed and wild populations sampled across the Mediterranean areas. Briefly, these retrieved datasets were from 12 DNA pools derived from farmed stocks of 7 countries and 12 DNA pools of wild stocks from 5 geographical locations. Each DNA pool was constituted by DNA obtained from 14 to 50 fish (Table S1) [7].

### 2.2. Sequencing, Alignment, and Variant Calling

For each group, pool-seq data (newly produced or retrieved from a previous study [7]) were trimmed with Trimmomatic v. 0.38 [27] with the following parameters: HEADCROP:9 and SLIDINGWINDOW:4:15 for the datasets produced from the investigated hatchery and MINLEN:36, CROP:148, SLIDINGWINDOW:4:15, and MINLEN:36 for the datasets retrieved from Peñaloza et al. 2021 [7]. Filtered paired reads were aligned to the gilthead seabream reference genome fSpaAur1.2 (NCBI accession no. GCA_900880675.1) using BWA 0.7.12 [28] with the *mem* function and standard options. Reads were filtered removing PCR duplicates and retaining only those with mapping quality of at least 30 using Samtools v1.10 [29]. Variant calling on the mapped reads was performed with the CRISP software using default parameters [30]. Only bi-allelic SNPs with quality >100 and depth ≥ 40 averaged across the datasets were considered.

The effect of each detected SNP was assessed with the Variant Effect Predictor [31] based on the gilthead seabream reference genome (GCA_900880675.1).

### 2.3. Population Genomic Analyses and Genome Annotation

Using the filtered SNPs from all DNA-pools, a Principal Component Analyses (PCA) was obtained with the “prcomp” function of R (https://www.R-project.org/ (accessed on 20 May 2022)).

Pairwise *F_ST_* values between DNA pools were calculated for each SNP located on assembled chromosomes, adapting the formula reported by Karlsson et al. 2007 [32]:

$$\begin{array}{c} Fst=\frac{\sum_{k=1}^{k}N_{k}}{\sum_{k=1}^{k}D_{k}}\\ N_{k}=p_{1}^{\left[k\right]}(q_{2}^{\left[k\right]}-q_{1}^{\left[k\right]})+p_{2}^{\left[k\right]}(q_{1}^{\left[k\right]}-q_{2}^{\left[k\right]})\\ D_{k}=p_{1}^{\left[k\right]}q_{2}^{\left[k\right]}+p_{2}^{\left[k\right]}q_{1}^{\left[k\right]} \end{array}$$

where 1, 2, *k* … *K* is the number of biallelic markers in the two populations and the variant allele for marker *k* has the frequencies $p_{1}^{\left[k\right]}$ and $p_{2}^{\left[k\right]}$ in the two populations. The global *F_ST_* of each pairwise comparison was retrieved by averaging the *F_ST_* calculated for each SNP. In this case, all DNA pools derived from wild populations and all DNA pools derived from farmed populations of the previous study [7] were analysed both as one wild and one farmed group and as separate populations.

For comparisons among the farmed Italian juvenile DNA pools, mean *F_ST_* values (m*F_ST_*) were calculated by averaging the SNP-based *F_ST_* in 50% overlapping window sizes of 500 kb, as already described in previous studies [12,13]. Here, windows with m*F*_ST_ values above the 99th percentile of the empirical distribution and with more than 200 SNPs were retained for further analyses.

Genes within these outlier regions were retrieved with bedtools [33] with the genome annotation file provided for fSpaAur1.2 in NCBI. Gene enrichment was performed among the genes in the outlier regions with PANTHER [34] and *Danio rerio* selected as the reference gene set. In this analysis, only Gene Ontology (GO) terms related to biological pathways with false discovery rate (FDR) <0.05 were considered as significantly enriched. Redundant GO terms were removed with REVIGO [35] considering the *D. rerio* database and applying SimRel as semantic similarity measure.

## 3. Results and Discussion

### 3.1. Population Genomic Parameters of Different Gilthead Seabream Stocks

**Figure 1:**
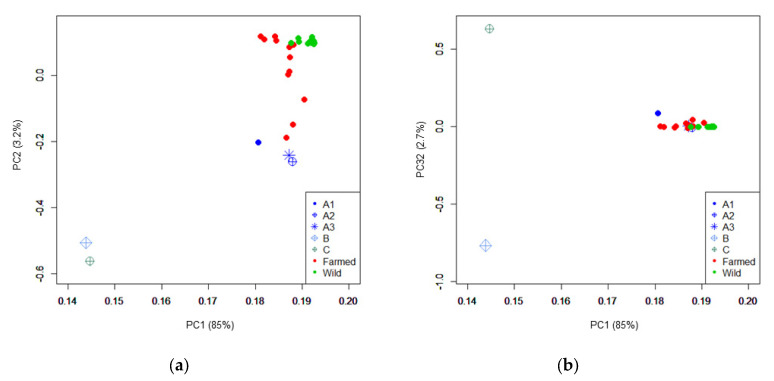
PCA obtained plots of the gilthead seabream populations and nuclei investigated in this study. The first three Principal Components (PC) are reported with the explained variation in parentheses. The three subpopulations of offspring derived from the same nucleus (A1, A2, and A3) are all labelled in blue but indicated with different marks. (**a**) PC1 1 and PC2; (**b**) PC1 and PC3.

The alignment of the sequencing data obtained from the five sequenced Italian farmed stocks to the *S. aurata* reference genome produced an average depth of coverage of 60× ± 1.73×. Detailed information on the sequencing depth of all DNA pools, including those retrieved from Peñaloza et al. 2021 [7], is reported in Table 1 and Table S1. The variant calling generated a total of 11,431,945 SNPs that were used to compute the PCA plots, shown in Figure 1. A close proximity between wild and farmed stocks was observed in agreement with what was reported by Peñaloza et al. 2021 [7]. This is probably due to the fact that breeding programs in this species involve periodic restocking or that, in most of the farmed populations, genetic programs are not yet well established. In Figure 1, the three DNA pools (A1, A2, and A3), derived from offspring of the A broodstock nucleus, are all close to the farmed DNA pools, whereas DNA pools B and C were distant from the rest of the pools and from each other. This distance is visible when considering both Component (C) 1 vs. C2 (Figure 1a) and C1 vs. C3 (Figure 1b).

This picture is confirmed by the consideration of the global *F_ST_* values. As expected due to their common origin from the same nucleus, the three pairwise comparisons between DNA pools A1, A2, and A3 showed low *F_ST_* values (<0.053). Also, they showed a low *F_ST_* value when compared with farmed and wild populations retrieved from a previous study (Table 2). This is confirmed also considering all the wild and farmed pools separately (Table S2). The latter populations were in turn quite close to each other (*F_ST_* = 0.010) suggesting that all these populations were not very differentiated from wild stocks. It is also worth mentioning that we analysed publicly available datasets by averaging information within two groups (farmed and wild) to reduce the genetic differentiation between the datasets. However, the closeness between nucleus A and these wild-related stocks may indicate that nucleus A experienced recent genetic introgression from wild populations or that neither selection pressure nor genetic drift drove substantial genetic differentiation from the original stocks. Nucleus A was quite different from both nuclei B and C. These two were also quite different from the other farmed and wild populations that we included in our study (Table 2 and Table S2). Nucleus B was also very distant from nucleus C (*F_ST_* = 0.405) suggesting very divergent selection trajectories and/or different origins.

**Figure 2:**
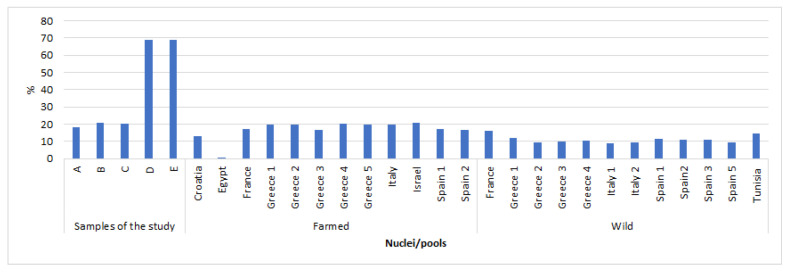
Proportion of monomorphic alleles observed in the different Italian gilthead seabream nuclei sequenced in this study and in farmed and wild populations retrieved from Peñaloza et al., 2021 [7].

These differences can also be seen when investigating the percentage of fixed alleles within each group (Figure 2). DNA pools A1, A2, and A3 had a similar percentage that ranged from 19 to 26%, in line with wild and farmed pools. The DNA pools with the highest percentage of monomorphic alleles were DNA pools B and C, both with 69% and an overlapping of SNPs of 68%, which confirms the genetic diversity of these pools underlined by the PCA analysis. The low genetic diversity observed in these nuclei may indicate a higher level of inbreeding than in nucleus A [36].

The control of inbreeding rate is very important when a selection program is implemented since inbreeding depression might negatively counterbalance any genetic progress. Estimates of inbreeding depression in fish have consistently shown that consanguineous progeny presents lower viability, less growth, lower reproductive performance, and less resistance to infections [37,38,39,40]. Since seabream is a species with high fecundity, nongenetic maternal effects and high mortality at early life stages can rapidly induce a loss of genetic variability if inbreeding is poorly considered in mating schemes [41].

Considering the common origin of the three DNA pools derived from nucleus A, in the subsequent genome-wide comparison analyses we combined these three subpopulations into one.

### 3.2. Genome-Wide Window-Based F_ST_ Analyses

**Figure 3:**
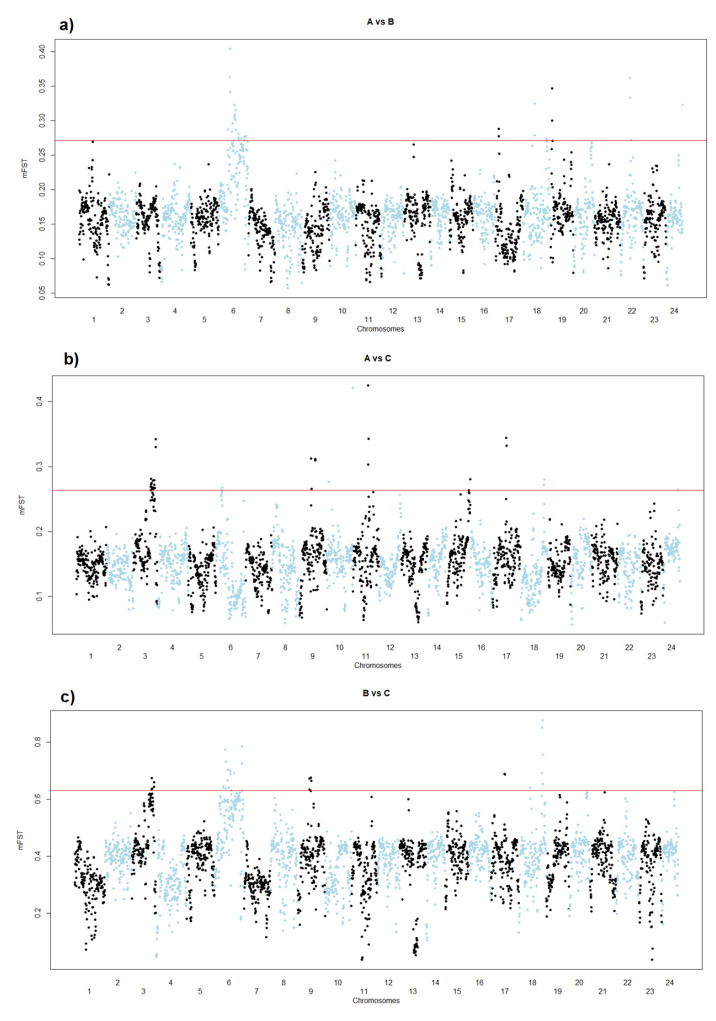
Manhattan plots of the m*FST* values observed in the pairwise analyses between the DNA pools constructed from the fry of the three different nuclei cultivated in the Italian hatchery: (**a**) DNA pool A vs. DNA pool B; (**b**) DNA pool A vs. DNA pool C; and (**c**) DNA pool B vs. DNA pool C.

**Figure 4:**
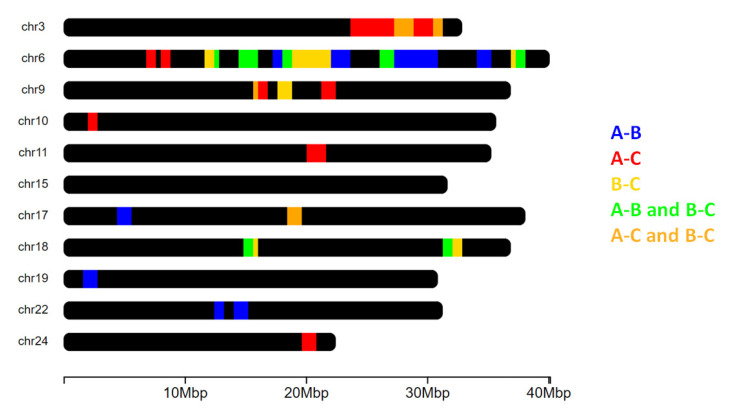
Overview of the chromosomes (chr) in which signatures of selection were detected. The x axis shows the chromosome length. The chromosomes that did not include any highlighted regions were not reported.

The results of the pairwise window-based *F_ST_* analyses between nuclei A, B, and C are graphically represented in the Manhattan plots of Figure 3 and are summarised in Figure 4. Tables S3–S5 reported all details of these comparisons with precise coordinates of the genomic regions that trespassed the defined thresholds and the genes included in those regions. From the comparison between nuclei A and B, a total of 14 genomic regions emerged. These outlier regions were located on six different chromosomes (6, 17, 18, 19, 22, and 24; Figure 3a and Table S3). The major differences between nuclei A and C (Figure 3b and Table S4) were detected in 11 regions located on 8 different chromosomes (3, 6, 9, 10, 11, 15, 17, 18, and 24) with the largest region spanning about 7.25 Mb of chromosome 3 (positions 23.75–31.0 Mb). The most relevant differences between nuclei B and C (Figure 3c and Table S5) were detected in 13 regions positioned on chromosomes 3, 6, 9, 17, and 18 with the largest region encompassing about 4.25 Mb on chromosome 6 (positions 17.75–22.00).

Considering the different pairwise comparisons, some partially overlapping regions were observed (Figure 3 and Figure 4): some were detected on chromosomes 3, 9, 17, and 18 for the comparisons between nuclei A and C and between nuclei B and C (Figure 4). Overlapping regions in the pairwise analyses, derived from nuclei A and B and between nuclei B and C, emerged on chromosomes 6, 17, and 18. Chromosome 6 contained a high number of signatures of selection derived from the pairwise comparisons A vs. B and B vs. C.

Some of the regions in the block 12–38 Mb of chromosome 6 were detected when comparing nuclei A and B and when comparing nuclei B and C, but they did not emerge when comparing nuclei A and C. These regions detected on chromosome 6 partially overlapped or were adjacent to regions that have been proposed as candidates for the lack of pigmentation defect in gilthead seabream [13]. This agrees with the fact that some of the fry that constituted nucleus A were depigmented or were carriers of the genetic defect causing depigmentation. Even if this phenotypic state of fry that composed nuclei B and C is unknown, from the present analyses we may postulate that nucleus C may have a higher genetic similarity with nucleus A in relation to the “depigmentation-carrier” and that nucleus B is the population that may have the lower proportion of this genetic defect.

### 3.3. Functional Annotation of Outliers F_ST_ Window Regions

#### 3.3.1. Impacts of SNPs

**Figure 5:**
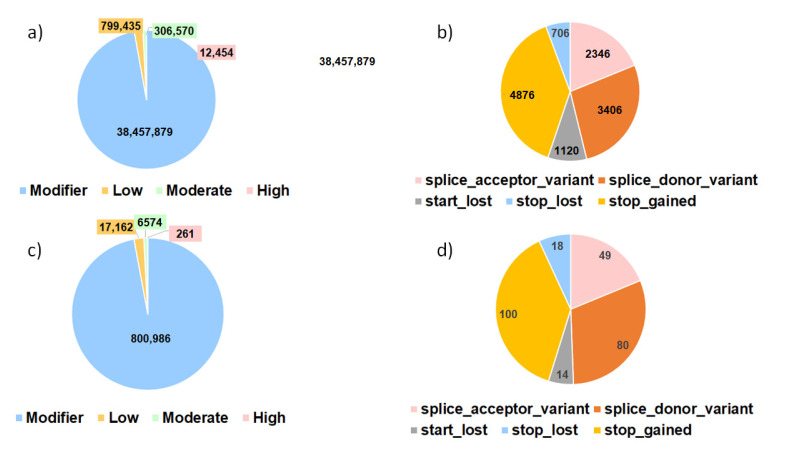
Proportion of variants detected (%) in the variant effect analyses for all the SNPs detected in the variant calling (**a**,**b**) and the variants identified in the detected outlier regions from the *F_ST_* analyses (**c**,**d**). (**a**,**c**) describe all the variants divided according to their predicted impact. Modifier: usually non-coding variants or variants affecting non-coding genes, where predictions are difficult or there is no evidence of impact. Moderate: a non-destructive variant that can change protein effectiveness. Low: assumed to be mostly harmless or unlikely to change protein behavior. High: the variant is assumed to have high (disruptive) impact in the protein, probably causing protein truncation, loss of function, or triggering nonsense mediated decay. (**b**,**d**) include only the proportions of the different predicted variations of the SNPs with high impact.

Variant effect analysis performed on the whole dataset is shown in Figure 5a,b. A total of 39,576,338 different effects have been detected: due to alternative splicing and gene overlapping along the genome, the same SNP location can have multiple predicted effects. The majority of the effects (97.17%) have been predicted as modifiers that might have no or very low impact. Other SNPs have been classified to have low impact (2.02%), moderate impact (0.77%), and high impact (0.07%) (Figure 5a). Among the 12,454 variations with high impact, most of them were catalogued as stop gained and stop lost and then splice variants (Figure 5b). In the regions with high *F_ST_* values, this proportion is unchanged, both considering the overall number (Figure 5c) and the distribution of the 261 SNPs with high impact (Figure 5d).

These variations with high impact were in 129 genes (Table S6). A few of the genes included in the high-impact *F_ST_* list had been previously linked with growth-related traits in other fish species. For example, fibronectin type III domain containing 5b (*FNDC5B*), which encodes for a subunit of the irisin protein, is known to regulate food intake, energy homeostasis, and glucose metabolism in teleosts [42,43]. This gene has been reported in QTL linked to head and body size in bighead carp *Hypophthalmichthys nobilis* [44,45]. Another gene related with head size in the same species is the solute carrier family 30 member 9 (*SLC30A9*) gene. Its encoded protein is involved in cellular zinc ion homeostasis and zinc ion transport. This gene has been proposed as a candidate for head length [45]. Another gene included in one of these regions was family with sequence similarity 135 member B (*FAM135B*), which encodes for a factor that promotes the secretion of the granulin growth factor, which has high expression in epithelial, immune, chondrocytes, and neuronal cells [46]. This gene has been reported to affect growth-related traits in livestock species [47] and in Chinese longsnout catfish [48]. In this catfish species, this gene has been suggested as a candidate for caudal peduncle depth, which is one of the parameters used to estimate fish growth [48].

#### 3.3.2. Overrepresentation of Biological Processes

The over-representation analyses revealed the enrichment of 27 GO terms in the regions that emerged in the comparison between nuclei A and B, 4 GO terms in the comparison between nuclei A and C, and 22 GO terms in the comparison between nuclei B and C. After filtering redundant GO terms, these numbers were reduced to a total of 24 GO terms in the contrast between nuclei A and B and 13 in the comparison between nuclei B and C (no redundant GO terms were detected in the analysis between nuclei A and C). All non-redundant GO terms are reported in Table S7. While comparisons of nucleus A vs. B and A vs. C detected GO terms mainly related to metabolism, comparison between nuclei B and C was enriched in GO terms related to morphogenesis and development. Even if the actual size of the fry that contributed to the different DNA pools is not known with precision and we do not have any phenotypic information available on the fish, this result may suggest that nuclei B and C are different in terms of growth potential. This may be of relevance as size in aquaculture can affect several steps of the selection, e.g., size “discard” at different developmental stages, and can also affect the final value of a single fish at market level.

Some of the genes containing high-impact variations were part of one or more GO terms derived from the previous enrichment analyses. For the genes detected in the comparison between nuclei A and B, it is worth mentioning the disintegrin and metalloprotease with thrombospondin type-1 motif member 9 *(ADAMTS9)*. This gene encodes for a cell-autonomous antiangiogenic metalloprotease that is involved in ovarian development [49]. In the enrichment analyses, this gene is included in several GO terms that are linked with metabolic processes and organ development. A multi-trait genome-wide association study in Nile tilapia associated variations in proximity to this gene with fillet trait [50]. Interleukin 1 receptor accessory protein-like 2 (*IL1RAPL2*), included in a genomic region that emerged in the comparison between nuclei A and B and present in the enriched gene list for cellular process (GO: 0009987), was suggested to be involved in the main QTL explaining 0.8% of variation for the acute hypoxia tolerance in a rainbow trout population [51]. The solute carrier family 4 member 8 (*SLC4A8*), having a large impact variation in the comparison between nuclei A and B, encodes for an electroneutral Na^+^-driven Cl^−^/HCO_3_^−^ exchanger whose expression in the gills seems to be reduced in salt water, as shown in Spotted seabass [52].

A region that emerged in the comparison between nuclei B and C contains variations with predicted high impact in the dual serine/threonine and tyrosine protein kinase (*D*STYK**) gene. This gene is a regulator of cell death, is expressed in multiple tissues, and was included in the GO terms linked with developmental processes. In zebrafish, a mutation on the *d*styk** gene is linked with spinal deformity [53].

One of the genes reported to have variations with high impact, present in one or more GO terms and shared in two comparisons (A vs. B and B vs. C), is the mediator of RNA polymerase II transcription subunit 12 homolog (*MED12*). This gene is involved in developmental processes and is part of the enriched GO terms linked to development and morphogenesis for the B vs. C comparison, for example, animal organ development (GO:0048513) and skeletal system morphogenesis (GO:0048705). It has also been proposed as a candidate gene explaining a QTL for body weight gain in rainbow trout [54].

## 4. Conclusions

*F_ST_* analyses underlined major genomic differences among the investigated gilthead seabream broodstock nuclei. Some of these differences highlighted genomic regions that contain genes involved in general metabolism and development; genes that have been detected in QTL for growth, size, and skeletal deformity; and adaptation to variation of oxygen level in other teleosts. The genomic diversity that was evidenced might be the results of the genetic histories of these nuclei, including the combined effect of directional selection, genetic drift, and bottleneck, that they might have experienced. Our results pointed out the need to control the genetic effects of breeding programmes in this species to avoid the negative effects of the reduction of genetic variability within the population with a consequent increase in the inbreeding level that, in turn, could lead to an increased frequency of deleterious alleles. Whole genome sequencing analyses might provide genomic pictures of breeding stocks that are useful to better design breeding strategies based on a whole genome-based information on the levels of within nucleus genetic variability and across nuclei genetic diversity so to enhance the production efficiency of the hatcheries.

## Figures and Tables

**Table 1:** Nuclei from the same hatchery investigated in this study, the number of fish used to constitute the DNA-pools, the overall number of high-quality reads utilised for the alignment, and depth of sequencing considering the alignment to the gilthead seabream reference genome.

Nuclei	No. of Fish	No. of Reads ^1^	Depth (×)
Italy A1	30	320,381,051	57.69
Italy A2	20	347,592,486	62.59
Italy A3	30	330,168,763	59.45
Italy B	30	341,904,089	61.57
Italy C	30	339,891,846	61.21

^1^ Number of filtered and aligned reads.

**Table 2:** Pairwise global *F_ST_* values for each of the groups and for the combined farmed and wild population retrieved from the previous study [7]. For further details about *F_ST_* comparisons see Table S2.

Nuclei/Populations	A1	A2	A3	B	C	Farmed	Wild
A1	0						
A2	0.053	0					
A3	0.052	0.032	0				
B	0.203	0.163	0.172	0			
C	0.191	0.162	0.169	0.405	0		
Farmed	0.05	0.04	0.04	0.163	0.166	0	
Wild	0.06	0.05	0.05	0.166	0.165	0.01	0

## Data Availability

Data are available in the following project number ENA: PRJEB33627.

## Supplementary Materials

The following supporting information can be downloaded at: https://www.mdpi.com/article/10.3390/genes14040839/s1. Table S1: Populations retrieved from Peñaloza et al. 2021 [7] included in this study, overall number of high-quality reads utilized for the alignment and depth of sequencing considering the alignment to the gilthead seabream reference genome. Table S2: Global *F_ST_* of the five pools under investigation against the single pools that composed the wild and farmed pools. Table S3: *F_ST_* regions of high divergence in the comparison between nuclei A and B. Reported data includes: chromosome, beginning of the region (start), end of the region (end), size of the region (size) and genes contained in the regions (genes). Table S4: *F_ST_* regions of high divergence in the comparison between nuclei A and C. Reported data includes: chromosome, beginning of the region (start), end of the region (end), size of the region (size) and genes contained in the regions (genes). Table S5: *F_ST_* regions of high divergence in the comparison between nuclei B and C. Reported data includes: chromosome, begin of the region (start), end of the region (end), size of the region (size) and genes contained in the regions (genes). Table S6: Variant effect predictor of high impact variation in the F*_ST_* regions. Table S7: Non-redundant Gene Ontology (GO) Term list for the different comparisons, including GO term code, GO term biological process name, fold enrichment, false discovery rate (FDR) value and genes included in the enrichment.
