# Supplementary material for: Whole Genome Sequencing Provides Information on the Genomic Architecture and Diversity of Cultivated Gilthead Seabream (Sparus aurata) Broodstock Nuclei
Source: Genes (Basel). 2023 Mar 30;14(4):839. doi: 10.3390/genes14040839 (PMC10137967; doi:10.3390/genes14040839)
Supplement: Supplementary file 1 [file genes-14-00839-s001.zip › genes-2264246-supplementary.pdf]

Supplementary tables

Whole Genome Sequencing Provides Information on the Genomic Architecture and Diversity of Cultivated Gilthead Seabream (*Sparus aurata*) Broodstock Nuclei

Francesca Bertolini [1,*], Anisa Ribani [1], Fabrizio Capoccioni [2], Luca Buttazzoni [2], Samuele Bovo [1], Giuseppina Schiavo [1], Massimo Caggiano [3], Max F. Rothschild [4] and Luca Fontanesi [1]

[1] Department of Agricultural and Food Sciences, Division of Animal Sciences, University of Bologna, Viale G. Fanin 46, 40127 Bologna, Italy; AR: anisa.ribani2@unibo.it; SB: Samuele.bovo@unibo.it; GS: Giuseppina.schiavo2@unibo.it; LF: luca.fontanesi@unibo.it
[2] Centro di ricerca "Zootecnia e Acquacoltura", Consiglio per la ricerca in agricoltura e l'analisi dell'economia agraria (CREA), 00198, Roma, Italy; FC: fabrizio.capoccioni@crea.gov.it; LB: luca.buttazzoni@crea.gov.it
[3] Panittica Italia Società Agricola Srl, 72016 Torre Canne di Fasano, Brindisi, Italy; maxcage29@gmail.com
[4] Department of Animal Science, Iowa State University, Ames, IA, 50011-3150, USA; mfrothsc@iastate.edu
* Correspondence: francesca.bertolini3@unibo.it

Table S1: Populations retrieved from [7] included in this study, overall number of high-quality reads utilized for the alignment and depth of sequencing considering the alignment to the gilthead seabream reference genome

Type of populations	Origin			
	**Country**	**N.animals**	**N. filtered and aligned reads**	**Depth (x)**
Farmed	Croatia	50	691,360,480	120.34
	Egypt	15	86,376,622	15.55
	France	50	598,335,029	107.74
	Greece 1	14	338,692,290	60.99
	Greece 2	13	309,693,828	55.77
	Greece 3	50	397,352,329	71.55
	Greece 4	50	428,129,407	77.09
	Greece 5	50	730,947,060	131.62
	Italy	50	329,332,200	59.30
	Israel	50	741,922,099	133.60
	Spain 1	50	642,865,590	115.76
	Spain 2	50	477,303,303	85.95
Wild	France	50	331,559,467	59.70
	Greece 1	50	393,380,659	70.84
	Greece 2	50	701,111,813	126.25
	Greece 3	50	592,892,137	106.76
	Greece 4	50	530,484,696	95.53
	Italy 1	50	650,086,955	117.06
	Italy 2	50	660,623,933	118.96
	Spain 1	50	688,175,668	123.92
	Spain2	50	696,493,010	125.42
	Spain 3	50	657,449,993	118.39
	Spain 5	50	670,932,580	120.82
	Tunisia	50	574,318,698	103.42

Table S2: Global F_{ST} of the five pools under investigation against the single pools that composed the wild and farmed pools

		A1	A2	A3	B	C
Farmed	Croatia	0.058	0.044	0.045	0.173	0.169
	Egypt	0.096	0.095	0.097	0.197	0.193
	France	0.060	0.041	0.043	0.178	0.170
	Greece 1	0.078	0.059	0.061	0.189	0.190
	Greece 2	0.078	0.063	0.065	0.193	0.192
	Greece 3	0.078	0.064	0.065	0.188	0.186
	Greece 4	0.089	0.074	0.076	0.198	0.198
	Greece 5	0.087	0.073	0.074	0.198	0.196
	Italy	0.056	0.041	0.038	0.181	0.176
	Israel	0.093	0.078	0.079	0.201	0.200
	Spain 1	0.079	0.065	0.067	0.190	0.187
	Spain 2	0.074	0.059	0.061	0.185	0.175
Wild	France	0.079	0.064	0.066	0.187	0.186
	Greece 1	0.071	0.057	0.058	0.177	0.176
	Greece 2	0.071	0.056	0.058	0.174	0.172
	Greece 3	0.069	0.055	0.056	0.173	0.172
	Greece 4	0.070	0.056	0.057	0.175	0.174
	Italy 1	0.068	0.053	0.055	0.172	0.170
	Italy 2	0.069	0.054	0.056	0.172	0.171
	Spain 1	0.071	0.057	0.058	0.176	0.183
	Spain2	0.070	0.055	0.058	0.176	0.174
	Spain 3	0.070	0.055	0.057	0.175	0.171
	Spain 5	0.068	0.053	0.055	0.172	0.182
	Tunisia	0.076	0.061	0.063	0.183	0.171

Table S3: F_{ST} regions of high divergence in the comparison between nuclei A and B. Reported data includes: chromosome, beginning of the region (start), end of the region (end), size of the region (size) and genes contained in the regions (genes).

chr	start	end	size (Mb)	genes
6	12,250,000	12,750,000	0.5	apcdd1l;asic1;bcdin3d;cers5;gpd1;LOC115582545;LOC115582924;LOC115582925;LOC115583137;LOC115583139;LOC115583141;LOC115583504;LOC115583733;LOC115583885;LOC115583932;LOC115583933;LOC115583934;LOC115583935;LOC115583936;LOC115584029;LOC115584031;manbal;ncoa5;ppp4r1;rab22a;smarcd1;src;trnat-ugu
6	14,750,000	15,750,000	1	alas1;amhr2;arhgap9;b9d2;c6h12orf10;col2a1;dctn2;ddit3;gli1;iars1;igsf8;itga7;kif5a;LOC115582574;LOC115582595;LOC115582596;LOC115582791;LOC115582956;LOC115582957;LOC115583161;LOC115583164;LOC115583209;LOC115583459;LOC115583461;LOC115583463;LOC115583495;LOC115583666;LOC115584128;LOC115584129;LOC115584148;mars1;nckap1l;npff;pde1b;poc1a;ppp1r1a;r3hdm2;rpl29;sp1;sp7;tarbp2;tespa1
6	17,500,000	18,750,000	1.25	abhd14b;brk1;c6h3orf18;c6h3orf67;cacna2d2;cplane2;edem1;h6pd;LOC115582615;LOC115582616;LOC115582618;LOC115582676;LOC115582677;LOC115582762;LOC115582782;LOC115582783;LOC115582810;LOC115582823;LOC115582824;LOC115582844;LOC115582880;LOC115582896;LOC115582992;LOC115583210;LOC115583408;LOC115583409;LOC115583426;LOC115583427;LOC115583428;LOC115583434;LOC115583464;LOC115583471;LOC115583472;LOC115583473;LOC115583474;LOC115583681;LOC115583682;LOC115583684;LOC115583685;LOC115583686;LOC115583723;LOC115583739;LOC115583775;LOC115583776;LOC115583777;LOC115583780;LOC115583849;LOC115583850;LOC115583940;LOC115583941;LOC115583942;LOC115583943;LOC115584108;mst1;rbm5;rrp9;stau1
6	20,500,000	23,500,000	3	adipor1;apof;atf1;b4galnt1;cav3;ccdc174;cdk2;cdk4;col7a1;crbn;cse1l;ctsz;eef1akmt3;eefsec;emc3;endou;erbb3;espl1;fancd2;fkbp11;gata2;grm2;ikzf4;irf6;LOC115582523;LOC115582563;LOC115582567;LOC115582585;LOC115582587;LOC115582590;LOC115582619;LOC115582620;LOC115582641;LOC115582679;LOC115582680;LOC115582700;LOC115582702;LOC115582710;LOC115582717;LOC115582744;LOC115582750;LOC115582812;LOC115582843;LOC115582887;LOC115582894;LOC115582907;LOC115582908;LOC115582909;LOC115582910;LOC115582911;LOC115582912;LOC115582913;LOC115582914;LOC115582987;LOC115582988;LOC115582989;slc4a8;LOC115583050;LOC115583060;LOC115583074;LOC115583075;LOC115583080;LOC115583082;LOC115583083;LOC115583104;LOC115583125;LOC115583127;LOC115583128;LOC115583129;LOC115583153;LOC115583155;LOC115583156;LOC115583204;LOC115583205;LOC115583342;LOC115583357;LOC115583358;LOC115583361;LOC115583363;LOC115583365;LOC115583376;LOC115583378;LOC115583380;LOC115583385;LOC115583387;LOC115583412;LOC115583413;LOC115583429;LOC115583432;LOC115583452;LOC115583453;LOC115583468;LOC115583469;LOC115583470;LOC115583486;LOC115583618;ankrd52;esyt1b;LOC115583651;LOC115583652;LOC115583662;LOC115583664;LOC115583673;LOC115583703;LOC115583708;LOC115583719;LOC115583732;LOC115583745;LOC115583760;LOC115583761;LOC115583782;LOC115583861;LOC115583862;LOC115583863;LOC115583864;LOC115583865;LOC115583867;LOC115583893;LOC115583897;LOC115583899;LOC115583902;LOC115583905;LOC115583919;LOC115583923;LOC115583945;LOC115583946;LOC115583947;LOC115583994;LOC115584026;LOC115584052;LOC115584054;LOC115584058;LOC115584059;LOC115584065;LOC115584070;LOC115584071;LOC115584081;LOC115584120;

				LOC115584133;LOC115584143;LOC115584152;mcrs1;mettl1;mmp19;nelfcd;npepl1;olfml3;os9;oxtr;pa2g4;parp3;pfdn5;prkag1;prkcd;prph;rabif;rad18;rft1;rps26;ruvbl1;smarcc2;smug1;srgap3;strip1;stx16;sumf1;suox;tatdn2;tex264;thumpd3;timeless;trnaf-gaa;tsfm;uroc1;usp4;vhl;wdr77;zc3h10
6	26,250,000	30,500,000	4.25	adamts9;aggf1;atxn7;camkv;cast;celf4;cidec;comt;cxxc1;dcaf12;dnah1;dnai1;drd5;ell2;eogt;glrx;gnl3l;hmces;id1;idh3b;kbtbd8;kiaa1328;LOC115582512;LOC115582532;LOC115582538;LOC115582569;LOC115582571;LOC115582572;LOC115582573;LOC115582579;LOC115582586;LOC115582601;LOC115582657;LOC115582658;LOC115582660;LOC115582667;LOC115582766;LOC115582767;LOC115582770;LOC115582828;LOC115582873;LOC115582882;LOC115582884;LOC115582918;LOC115582946;LOC115582949;myh7ba;LOC115583067;LOC115583070;LOC115583071;LOC115583077;LOC115583079;LOC115583117;LOC115583118;LOC115583119;LOC115583120;LOC115583122;LOC115583147;LOC115583167;LOC115583168;LOC115583176;LOC115583179;LOC115583180;LOC115583201;LOC115583329;LOC115583349;LOC115583354;LOC115583399;LOC115583400;LOC115583401;LOC115583402;LOC115583403;LOC115583411;LOC115583490;LOC115583492;LOC115583501;LOC115583502;LOC115583507;LOC115583550;LOC115583553;LOC115583562;LOC115583580;LOC115583593;LOC115583594;LOC115583610;LOC115583611;LOC115583613;LOC115583646;LOC115583647;LOC115583648;LOC115583689;LOC115583699;LOC115583701;LOC115583755;LOC115583767;LOC115583793;cdh4;LOC115583846;LOC115583851;LOC115583855;LOC115583879;LOC115583909;LOC115583910;LOC115583921;LOC115583925;LOC115583926;LOC115583927;LOC115583939;LOC115583992;dkey-202e22.2;LOC115584036;LOC115584049;LOC115584097;LOC115584098;LOC115584107;LOC115584109;LOC115584130;LOC115584145;LOC115584150;LOC115584151;LOC115584156;LOC115584191;LOC115584192;LOC115584196;lrig1;lyar;magi1;mon1a;mst1r;mustn1;myorg;ndrg3;nop56;nsg1;nudt2;otop1;pcsk1;prickle2;psmd6;ptpra;pxn;r3hcc1;rae1;rfesd;rhobtb3;rplp0;rpn1;sfmbt1;sla2;slc25a26;slc2a9;stx18;suclg2;sulf2;synpr;tada3;tfe3;thoc7;tmem128;tmem233;tmf1;tpgs2;traip;trnae-cuc;trnaf-gaa;trnaq-cug;trnaq-uug;trnar-acg;trpc4apa;ubap1;ubap2;cxxc1b;ube2r2;wdr1;grm6b;zbtb49;ndrg3a
6	34,500,000	35,250,000	0.75	barx1;cass4;cstf1;fam120a;LOC115583818;LOC115583819;fbln2;LOC115583825;LOC115583828;LOC115583830;LOC115583833;LOC115583834;LOC115583837;pdrg1;phf2;ptpdc1
6	37,250,000	37,750,000	0.5	helz2;LOC115582790;LOC115582792;LOC115582892;aldh1l1;LOC115583158;LOC115583211;LOC115583390;LOC115583769;LOC115583953;LOC115584023;plekha6;

17	4,500,000	5,250,000	0.75	cnot10;hace1;kpna5;LOC115567168;LOC115567171;LOC115567172;LOC115567173;LOC115567458;LOC115567467;LOC115567468;LOC115567470;LOC115567472;LOC115567473;LOC115567474;LOC115567479;LOC115567481;LOC115567485;LOC115567486;LOC115567487;LOC115567488;LOC115567489;LOC115567686;LOC115567687;LOC115567688;LOC115567842;rwdd1;zup1
18	15,250,000	16,000,000	0.75	fmr1;hdac3;LOC115568236;LOC115568441;LOC115568557;LOC115568808;LOC115568894;LOC115568898;rab33a
18	31,500,000	32,250,000	0.75	btk;enox2;gla;LOC115568228;LOC115568375;LOC115568503;LOC115568505;LOC115568506;LOC115568934;slc4a11;LOC115568979;LOC115568980;mars2;med12;rpl36a;sfxn5;smyd5;timm8a
19	1,750,000	2,500,000	0.75	LOC115569643;LOC115569644;LOC115569645;LOC115569646;LOC115569647;LOC115569714;LOC115570178;LOC115570179;LOC115570180;LOC115570449;LOC115570451
22	12,500,000	13,250,000	0.75	abracl;arhgap11b;aven;clu;garem2;grem1;hadhb;heca;knl1;LOC115573825;LOC115573830;LOC115573881;LOC115573992;LOC115573995;LOC115574060;LOC115574061;LOC115574193;LOC115574342;LOC115574485;LOC115574615;mtif3;nudt14;reps1;scara3;scg5
22	14,500,000	15,000,000	0.5	bpnt1;c22h12orf57;cnr1;extl3;LOC115573515;LOC115574247;LOC115574251;LOC115574380;LOC115574382;LOC115574442;LOC115574443;LOC115574518;LOC115574520;LOC115574523;LOC115574524;LOC115574610;LOC115574614;ptprk;rars2;rngtt;saysd1;slc35a1
24	22,250,000	22,750,000	0.5	LOC115576605;LOC115576613;LOC115576635;LOC115576647;LOC115576649;LOC115576664;LOC115576712;LOC115576725;LOC115576727;LOC115576966;LOC115576967;LOC115576968

Table S4: F_{ST} regions of high divergence in the comparison between nuclei A and C. Reported data includes: chromosome, beginning of the region (start), end of the region (end), size of the region (size) and genes contained in the regions (genes).

chr	start	end	size (Mb)	genes
3	23,750,000	31,000,000	7.25	abcf1;adck5;agr2;bzw2;c3h5orf49;c3h6orf136;chtop;col22a1;cpsf1;ctdp1;eif3i;fam135b;gatad2b;ilf2;irx1;kcnk9;khdrbs3;kpna6;zgc:91944;LOC115577673;LOC115577688;LOC115577765;LOC115577776;LOC115577793;LOC115577801;LOC115577876;LOC115578217;LOC115578325;LOC115578329;LOC115578353;LOC115578354;LOC115578360;LOC115578361;LOC115578363;LOC115578364;LOC115578372;LOC115578389;LOC115578394;LOC115578396;LOC115578397;LOC115578402;LOC115578409;LOC115578410;LOC115578412;LOC115578413;LOC115578414;LOC115578417;LOC115578431;LOC115578450;LOC115578457;LOC115578467;LOC115578481;LOC115578486;LOC115578492;LOC115578508;LOC115578509;LOC115578510;LOC115578534;LOC115578546;LOC115578547;LOC115578568;LOC115578569;LOC115578570;LOC115578572;LOC115578573;LOC115578574;arid1ab;LOC115578681;LOC115578682;grb10a;LOC115578711;LOC115578718;LOC115578719;LOC115578720;LOC115578721;LOC115578722;LOC115578725;LOC115578739;LOC115578793;LOC115578806;fndc5b;LOC115578808;LOC115578809;LOC115578810;LOC115578821;LOC115578827;ptprua;LOC115578863;LOC115578897;LOC115578915;LOC115578921;LOC115578922;LOC115578929;LOC115578930;LOC115578932;LOC115578961;LOC115578965;LOC115578972;LOC115578979;LOC115578981;LOC115578983;LOC115578988;LOC115579055;rbbp7;LOC115579058;LOC115579059;LOC115579084;adcy2b;LOC115579115;LOC115579118;LOC115579127;LOC115579136;LOC115579144;LOC115579145;LOC115579146;LOC115579148;LOC115579149;LOC115579153;LOC115579154;LOC115579162;LOC115579163;LOC115579186;LOC115579195;LOC115579196;LOC115579232;LOC115579263;LOC115579303;LOC115579309;LOC115579369;LOC115579372;LOC115579399;LOC115579400;LOC115579415;LOC115579425;LOC115579426;LOC115579451;LOC115579460;LOC115579470;LOC115579474;LOC115579476;LOC115579503;LOC115579553;LOC115579554;LOC115579555;LOC115579556;LOC115579560;LOC115579561;LOC115579563;LOC115579570;LOC115579594;LOC115579595;LOC115579601;LOC115579602;LOC115579604;LOC115579607;LOC115579608;LOC115579611;med10;mfsd2a;mrps18b;mturn;ndufs6;ngly1;nkiras1;Note=The;nr1d2;nsun2;nudc;oxsm;parp10;pex11b;pkib;ppp1r10;rarb;rpl14;rpl15;sf3a3;slc66a2;snapin;srd5a1;srfbp1;st3gal1;tent4a;tert;thrb;top2b;topaz1;trappc9;trim71;trit1;tspan13;txnip;txnl4a;ube2ql1;yars1
6	7,000,000	7,500,000	0.5	blcap;LOC115582671;LOC115582672;LOC115582673;LOC115582842;LOC115582867;chd6;LOC115582955;ripor3;LOC115583557;LOC115583741;LOC115583743;mcm2;pigu;podxl2;psmf1;rbl1;tmem74b
6	8,250,000	8,750,000	0.5	aspn;ccdc71;cenpp;cept1;dram2;ecm2;hgh1;ippk;LOC115582539;LOC115582878;LOC115583443;LOC115583545;LOC115583574;LOC115583602;LOC115583604;LOC115584089;LOC115584122;nisch;nol8;ogn;rbbp5;stab1
9	16,000,000	16,750,000	0.75	acp6;bcl9;cd58;chaf1b;dop1b;fstl1;gja5;gja8;gpr156;gsk3b;igsf3;kcne1;klhl6;LOC115587460;LOC115587572;LOC115587587;LOC115587692;LOC115587699;LOC115587701;LOC115587784;LOC115587787;LOC115587965;LOC115588161;LOC115588281;LOC115588340;LOC115588378;LOC115588409;LOC115588412;LOC115588413;LOC115588414;LOC115588431;LOC115588432;LOC115588433;LOC115588434;LOC115588535;LOC115588615;lrrc58;maats1;morc3;nr1i2;rpe;slc5a3

9	21,500,000	22,250,000	0.75	bin1;cflar;dip2a;ercc1;fam126b;LOC115587442;LOC115587507;LOC115587920;LOC115587921;LOC115587922;ptprna;LOC115588188;LOC115588189;LOC115588277;LOC115588290;LOC115588569;LOC115588570;LOC115588572;LOC115588573;LOC115588576;LOC115588577;LOC115588605;LOC115588606;LOC115588607;LOC115588609;LOC115588610;LOC115588618;LOC115588619;map3k13;pofut2;prmt2;snx4;tmem169;trpm2;xrcc5
10	2,250,000	2,750,000	0.5	LOC115589993;LOC115590010;LOC115590019;LOC115590028;LOC115590030;LOC115590046;LOC115590049;LOC115590050;LOC115590069;LOC115590096;LOC115590103;LOC115590105;LOC115590107;LOC115590114;LOC115590121;LOC115590122;LOC115590132;LOC115590348;LOC115590349;LOC115590350;LOC115590351;LOC115590352;LOC115590354
11	20,500,000	21,500,000	1	ccdc124;cluap1;LOC115590926;LOC115590950;LOC115591005;LOC115591006;LOC115591014;LOC115591020;ptprsa;LOC115591422;LOC115591555;LOC115591623;LOC115591624;LOC115591626;jak3;LOC115592047;mast3b;ndufa7;slc5a5;uhrf1;mast3
15	31,750,000	32,250,000	0.5	bmpr1a;clhc1;rps27a;rtn4
17	18,500,000	19,250,000	0.75	aplp1;has1;hspb6;igflr1;kcnn4;kirrel2;kmt2b;lin37;LOC115567082;LOC115567083;LOC115567084;LOC115567085;LOC115567086;LOC115567088;LOC115567089;LOC115567090;LOC115567094;LOC115567095;LOC115567096;LOC115567097;LOC115567102;LOC115567103;LOC115567105;LOC115567106;LOC115567107;LOC115567108;LOC115567109;LOC115567110;LOC115567111;LOC115567115;LOC115567116;LOC115567117;LOC115567119;LOC115567121;LOC115567124;LOC115567125;LOC115567126;LOC115567427;LOC115567573;LOC115567574;LOC115567575;LOC115567610;LOC115567948;LOC115567979;nphs1;pilrb;plaur;proser3;psenen;smg9;spaca6;zbtb32
18	31,750,000	32,500,000	0.75	atp8a1;bend4;clnk;enox2;grxcr1;gsr;hs3st1;LOC115568228;LOC115568245;LOC115568462;LOC115568934;slc4a11;LOC115568979;LOC115568980;sfxn5;shisa3;slc30a9;smyd5;znf518b
24	20,000,000	20,500,000	0.5	LOC115576539;LOC115576541;LOC115576543;LOC115576544;LOC115576545;LOC115576624;LOC115576699;LOC115576700;LOC115576701;LOC115576704;LOC115576708;LOC115576711;LOC115576713;LOC115576714;LOC115576720;LOC115576721;LOC115576723;LOC115576728;LOC115576986

Table S5: F_{ST} regions of high divergence in the comparison between nuclei B and C. Reported data includes: chromosome, begin of the region (start), end of the region (end), size of the region (size) and genes contained in the regions (genes)

chr	start	end	size (Mb)	genes
3	27,000,000	28,500,000	1.5	col22a1;fam135b;kcnk9;LOC115577765;LOC115577776;LOC115578360;LOC115578389;LOC115578457;LOC115578508;arid1ab;LOC115578711;LOC115578915;LOC115578921;LOC115578922;LOC115578972;LOC115578983;LOC115578988;LOC115579084;LOC115579145;LOC115579146;LOC115579303;LOC115579372;LOC115579425;LOC115579426;LOC115579503;nudc;pkib;trappc9
3	30,250,000	31,000,000	0.75	abcf1;agr2;bzw2;c3h6orf136;chtop;gatad2b;ilf2;LOC115577673;LOC115578325;LOC115578329;LOC115578353;LOC115578354;LOC115578396;LOC115578397;LOC115578681;LOC115579196;LOC115579399;LOC115579400;LOC115579601;LOC115579602;LOC115579607;mrps18b;ngly1;oxsm;pex11b;ppp1r10;rarb;snapin;top2b;tspan13;txnip
6	12,000,000	12,750,000	0.75	apcdd1l;asic1;bcdin3d;cers5;gpd1;LOC115582545;LOC115582593;LOC115582606;LOC115582924;LOC115582925;LOC115583137;LOC115583139;LOC115583141;LOC115583504;LOC115583733;LOC115583734;LOC115583735;LOC115583885;LOC115583932;LOC115583933;LOC115583934;LOC115583935;LOC115583936;LOC115584029;LOC115584031;manbal;ncoa5;ppp4r1;rab22a;smarcd1;src;trnat-ugu
6	14,750,000	15,750,000	1	alas1;amhr2;arhgap9;b9d2;c6h12orf10;col2a1;dctn2;ddit3;gli1;iars1;igsf8;itga7;kif5a;LOC115582574;LOC115582595;LOC115582596;LOC115582791;LOC115582956;LOC115582957;LOC115583161;LOC115583164;LOC115583209;LOC115583459;LOC115583461;LOC115583463;LOC115583495;LOC115583666;LOC115584128;LOC115584129;LOC115584148;mars1;nckap1l;npff;pde1b;poc1a;ppp1r1a;r3hdm2;rpl29;sp1;sp7;tarbp2;tespa1
6	15,000,000	15,500,000	0.5	amhr2;c6h12orf10;col2a1;dctn2;ddit3;igsf8;kif5a;LOC115582574;LOC115582595;LOC115582596;LOC115582791;LOC115582956;LOC115582957;LOC115583459;LOC115583461;LOC115583463;mars1;nckap1l;pde1b;ppp1r1a;sp1;sp7;tarbp2

6	17,750,000	22,000,000	4.25	abhd14a;acy1;apof;b4galnt1;bap1;brk1;bysl;c6h1orf159;c6h1orf74;c6h3orf67;capzb;card19;cav3;ccdc174;ccdc36;celsr2;col7a1;cplane2;crbn;crocc;cse1l;dstyk;edem1;eefsec;eif4enif1;emc3;erbb3;erc2;fancd2;fblim1;fbxo42;fkbp11;fkbp5;fndc10;gata2;grm2;h6pd;hdhd3;ikzf4;irf6;kbtbd12;LOC115582523;LOC115582554;LOC115582555;LOC115582557;LOC115582567;LOC115582585;LOC115582587;LOC115582590;LOC115582602;LOC115582614;LOC115582615;LOC115582616;LOC115582618;LOC115582641;LOC115582664;LOC115582676;LOC115582677;LOC115582700;LOC115582703;LOC115582712;LOC115582727;LOC115582728;LOC115582750;LOC115582762;LOC115582782;LOC115582783;LOC115582810;LOC115582812;LOC115582823;LOC115582824;LOC115582826;LOC115582843;LOC115582844;LOC115582847;LOC115582880;LOC115582887;LOC115582896;LOC115582897;LOC115582901;LOC115582903;LOC115582904;LOC115582969;LOC115582992;LOC115582993;LOC115582994;LOC115582995;LOC115583000;LOC115583003;LOC115583004;LOC115583007;LOC115583009;LOC115583012;LOC115583014;LOC115583017;LOC115583019;LOC115583020;LOC115583023;LOC115583024;LOC115583025;LOC115583027;LOC115583030;LOC115583032;LOC115583035;LOC115583041;LOC115583042;LOC115583060;LOC115583074;LOC115583075;LOC115583080;LOC115583082;LOC115583083;LOC115583093;LOC115583096;LOC115583098;LOC115583099;LOC115583100;LOC115583104;LOC115583125;LOC115583127;LOC115583128;LOC115583129;LOC115583142;LOC115583143;LOC115583145;LOC115583146;LOC115583153;LOC115583155;LOC115583156;LOC115583189;LOC115583190;LOC115583191;LOC115583193;LOC115583195;LOC115583210;LOC115583342;LOC115583355;LOC115583408;LOC115583409;LOC115583412;LOC115583413;LOC115583426;LOC115583429;LOC115583452;LOC115583453;LOC115583464;LOC115583471;LOC115583472;LOC115583473;LOC115583474;LOC115583486;LOC115583560;LOC115583561;LOC115583609;LOC115583662;LOC115583664;LOC115583681;LOC115583682;LOC115583684;LOC115583685;LOC115583686;LOC115583708;LOC115583710;LOC115583713;LOC115583723;LOC115583739;LOC115583745;LOC115583761;LOC115583775;LOC115583776;LOC115583777;LOC115583780;LOC115583782;LOC115583790;LOC115583849;LOC115583850;LOC115583881;LOC115583883;LOC115583893;LOC115583897;LOC115583899;LOC115583901;LOC115583902;LOC115583919;LOC115583940;LOC115583941;LOC115583942;LOC115583943;LOC115583983;LOC115584034;LOC115584070;LOC115584071;LOC115584081;LOC115584088;LOC115584108;LOC115584113;LOC115584114;LOC115584115;LOC115584136;LOC115584137;LOC115584138;LOC115584144;LOC115584152;LOC115584194;med20;mfap2;mrpl20;nbl1;nckipsd;ndufaf3;necap2;Note=The;nuak2;os9;oxtr;p4htm;pa2g4;parp3;patz1;ppil1;prkag1;prkcd;rad18;rbm5;rft1;rps26;rrp9;ruvbl1;sfi1;sirt4;slc25a20;slc25a34;srgap3;srsf3;ssu72;stau1;strip1;szrd1;tatdn2;tex264;thumpd3;tmcc2;tmem43;tmem88b;tomm6;trnaf-gaa;trub2;tspo;uroc1;usp4;usp49;vhl;vwa1;wdr77
6	26,250,000	27,000,000	0.75	aggf1;celf4;dnai1;kiaa1328;LOC115582884;LOC115582918;LOC115583147;LOC115583179;LOC115583180;LOC115583507;LOC115583550;LOC115583553;LOC115583646;LOC115583767;LOC115583855;LOC115583925;LOC115583926;LOC115584130;myorg;nsg1;pxn;r3hcc1;rplp0;stx18;tmem233;tpgs2
6	37,000,000	38,000,000	1	helz2;LOC115582699;LOC115582786;LOC115582790;LOC115582792;LOC115582892;aldh1l1;LOC115583158;LOC115583211;LOC115583390;LOC115583508;LOC115583524;LOC115583769;LOC115583891;LOC115583953;LOC115584023;LOC115584056;nfasc;plekha6;zc3h11a

9	15,750,000	16,500,000	0.75	acp6;bcl9;btla;chaf1b;dop1b;fam155a;fstl1;gja5;gja8;gpr156;gsk3b;kcne1;LOC115587460;LOC115587467;LOC115587468;LOC115587469;LOC115587512;LOC115587558;LOC115587572;LOC115587601;LOC115587692;LOC115587784;LOC115587787;LOC115587965;LOC115588160;LOC115588161;LOC115588281;LOC115588345;LOC115588378;LOC115588409;LOC115588412;LOC115588413;LOC115588414;LOC115588517;LOC115588615;LOC115588645;lrrc58;maats1;morc3;nr1i2;pdzk1;slc5a3
9	18,000,000	18,750,000	0.75	adam23;agps;atf2;atp5mc3;chn1;chrna1;cir1;dock9;evx2;fam237a;gpr1;gpr155;hnrnpa3;hoxd10;hoxd11;hoxd3;hoxd4;hoxd9;ino80d;lnpk;LOC115587472;LOC115587908;stk24;LOC115588208;LOC115588210;LOC115588211;LOC115588710;ndufs1;nfe2l2;ola1;osbpl6;pde11a;prkra;scrn3;sp9
17	18,500,000	19,250,000	0.75	aplp1;has1;hspb6;igflr1;kcnn4;kirrel2;kmt2b;lin37;LOC115567082;LOC115567083;LOC115567084;LOC115567085;LOC115567086;LOC115567088;LOC115567089;LOC115567090;LOC115567094;LOC115567095;LOC115567096;LOC115567097;LOC115567102;LOC115567103;LOC115567105;LOC115567106;LOC115567107;LOC115567108;LOC115567109;LOC115567110;LOC115567111;LOC115567115;LOC115567116;LOC115567117;LOC115567119;LOC115567121;LOC115567124;LOC115567125;LOC115567126;LOC115567427;LOC115567573;LOC115567574;LOC115567575;LOC115567610;LOC115567948;LOC115567979;nphs1;pilrb;plaur;proser3;psenen;smg9;spaca6;zbtb32
18	15,500,000	16,000,000	0.5	fmr1;hdac3;LOC115568236;LOC115568441;LOC115568557;LOC115568808;LOC115568894;LOC115568898;rab33a
18	31,500,000	33,000,000	1.5	atp8a1;bend4;bod1l1;btk;clnk;cops6;enox2;fbxl12;gla;gpc2;grxcr1;gsr;hs3st1;LOC115568228;LOC115568245;LOC115568328;LOC115568375;LOC115568392;LOC115568462;LOC115568503;LOC115568505;LOC115568506;LOC115568658;LOC115568659;LOC115568660;LOC115568661;LOC115568662;LOC115568663;LOC115568664;LOC115568665;LOC115568666;LOC115568667;LOC115568668;LOC115568669;LOC115568723;LOC115568724;LOC115568725;LOC115568726;LOC115568729;LOC115568735;LOC115568737;LOC115568738;LOC115568739;LOC115568740;LOC115568885;LOC115568934;LOC115568977;LOC115568979;LOC115568980;LOC115569169;LOC115569171;mars2;mcm7;med12;nkx3-2;rab28;rpl36a;sfxn5;shisa3;slc30a9;smyd5;stag3;timm8a;trnal-aag;znf518b

Table S6: Variant effect predictor of high impact variation in the F_{ST} regions

chr	pos	Allele	Gene	Gene symbol	Consequence
3	23,786,331	A	ENSSAUG00010005037	slc6a18	splice_acceptor_variant
3	23,793,969	T	ENSSAUG00010005043	tert	splice_acceptor_variant
3	23,801,486	C	ENSSAUG00010005061	RBBP7	stop_lost
3	23,865,408	C	ENSSAUG00010005084	yars1	stop_lost
3	24,009,698	T	ENSSAUG00010006540	fam49al	splice_donor_variant
3	24,136,370	T	ENSSAUG00010006618	TRAK1	stop_gained
3	24,241,732	T	ENSSAUG00010006635	-	stop_gained
3	24,286,730	T	ENSSAUG00010006641	-	splice_acceptor_variant
3	24,506,671	A	ENSSAUG00010006657	-	stop_gained
3	24,555,500	T	ENSSAUG00010006658	-	stop_gained
3	24,556,819	T	ENSSAUG00010006658	-	stop_gained
3	24,572,859	G	ENSSAUG00010006661	-	splice_donor_variant
3	24,640,520	T	ENSSAUG00010006671	grb10a	stop_gained
3	24,640,600	A	ENSSAUG00010006671	grb10a	stop_gained
3	24,687,470	A	ENSSAUG00010006705	trioa	splice_donor_variant
3	24,687,470	A	ENSSAUG00010006705	trioa	splice_donor_variant
3	24,795,307	A	ENSSAUG00010006753	adcy2b	stop_gained
3	24,795,307	A	ENSSAUG00010006753	adcy2b	stop_gained
3	24,795,307	A	ENSSAUG00010006753	adcy2b	stop_gained
3	24,795,307	A	ENSSAUG00010006753	adcy2b	stop_gained
3	24,853,473	T	ENSSAUG00010008753	tent4a	splice_acceptor_variant
3	25,532,913	A	ENSSAUG00010008863	fndc5b	stop_gained
3	25,585,504	A	ENSSAUG00010008865	-	stop_gained
3	26,129,353	G	ENSSAUG00010000814	zgc:91944	stop_lost
3	26,146,149	T	ENSSAUG00010000822	-	stop_lost
3	26,555,336	T	ENSSAUG00010000978	cpsf1	splice_donor_variant
3	26,566,540	T	ENSSAUG00010000978	cpsf1	splice_donor_variant
3	26,566,540	T	ENSSAUG00010000978	cpsf1	splice_donor_variant
3	26,566,540	T	ENSSAUG00010000978	cpsf1	splice_donor_variant
3	26,572,779	A	ENSSAUG00010007232	-	splice_acceptor_variant
3	26,572,780	G	ENSSAUG00010007232	-	splice_acceptor_variant
3	26,572,813	G	ENSSAUG00010007232	-	splice_donor_variant
3	27,064,623	G	ENSSAUG00010007238	FAM135B	splice_acceptor_variant
3	27,630,256	C	ENSSAUG00010007291	arid1ab	stop_gained
3	27,657,190	A	ENSSAUG00010007291	arid1ab	splice_donor_variant
3	28,540,731	A	ENSSAUG00010008010	-	stop_gained
3	28,704,907	T	ENSSAUG00010008056	ptprua	stop_gained
3	28,763,272	G	ENSSAUG00010008056	ptprua	splice_donor_variant
3	28,792,771	C	ENSSAUG00010008056	ptprua	stop_gained
3	28,792,771	C	ENSSAUG00010008056	ptprua	stop_gained
3	28,812,844	T	ENSSAUG00010008056	ptprua	start_lost
3	30,299,861	A	ENSSAUG00010014936	-	stop_gained
3	30,993,630	A	ENSSAUG00010005142	-	splice_donor_variant

6	7,156,030	A	ENSSAUG00010014707	chd6	stop_gained
6	7,156,030	A	ENSSAUG00010014707	chd6	stop_gained
6	7,156,030	A	ENSSAUG00010014707	chd6	stop_gained
6	7,156,030	A	ENSSAUG00010014707	chd6	stop_gained
6	7,156,030	A	ENSSAUG00010014707	chd6	stop_gained
6	7,156,692	T	ENSSAUG00010014707	chd6	splice_acceptor_variant
6	7,156,692	T	ENSSAUG00010014707	chd6	splice_acceptor_variant
6	7,156,692	T	ENSSAUG00010014707	chd6	splice_acceptor_variant
6	7,318,187	T	ENSSAUG00010015434	ripor3	splice_acceptor_variant
6	7,331,908	T	ENSSAUG00010015434	ripor3	stop_gained
6	7,348,841	A	ENSSAUG00010015438	-	stop_gained
6	7,428,322	C	ENSSAUG00010015485	rbl1	splice_acceptor_variant
6	7,429,621	A	ENSSAUG00010015485	rbl1	splice_donor_variant
6	7,434,847	G	ENSSAUG00010015485	rbl1	splice_acceptor_variant
6	7,434,939	T	ENSSAUG00010015485	rbl1	splice_donor_variant
6	7,434,978	A	ENSSAUG00010015485	rbl1	stop_gained
6	7,435,063	T	ENSSAUG00010015485	rbl1	splice_donor_variant
6	7,438,846	A	ENSSAUG00010015485	rbl1	stop_gained
6	7,470,142	A	ENSSAUG00010016301	si:ch211-193l2.10	splice_acceptor_variant
6	7,487,795	A	ENSSAUG00010016308	-	splice_acceptor_variant
6	7,487,795	A	ENSSAUG00010016308	-	splice_acceptor_variant
6	7,487,795	A	ENSSAUG00010016308	-	splice_acceptor_variant
6	7,487,795	A	ENSSAUG00010016308	-	splice_acceptor_variant
6	8,384,418	T	ENSSAUG00010022893	-	stop_gained
6	8,424,704	G	ENSSAUG00010022897	rbbp5	stop_lost
6	8,531,912	G	ENSSAUG00010022904	cenpp	splice_donor_variant
6	8,543,035	T	ENSSAUG00010022915	nol8	stop_lost
6	8,660,158	A	ENSSAUG00010022933	atp2b4	splice_donor_variant
6	8,678,277	A	ENSSAUG00010022933	atp2b4	splice_acceptor_variant
6	8,728,969	T	ENSSAUG00010023294	cept1b	stop_gained
6	8,731,610	T	ENSSAUG00010023294	cept1b	splice_acceptor_variant
6	12,309,176	T	ENSSAUG00010000584	slc12a5a	splice_donor_variant
6	12,309,176	T	ENSSAUG00010000584	slc12a5a	splice_donor_variant
6	12,502,889	G	ENSSAUG00010000663	cyp24a1	start_lost
6	12,714,565	A	ENSSAUG00010003785	smarcd1	splice_donor_variant
6	15,115,037	G	ENSSAUG00010024021	soat2	splice_acceptor_variant
6	15,221,722	C	ENSSAUG00010024036	AMHR2	start_lost
6	15,423,721	T	ENSSAUG00010024435	NCKAP1L	stop_gained
6	18,091,491	G	ENSSAUG00010020707	uba7	start_lost
6	18,989,322	A	ENSSAUG00010020171	slc6a22.2	splice_acceptor_variant
6	19,679,068	T	ENSSAUG00010024949	il17rc	splice_donor_variant
6	19,741,584	A	ENSSAUG00010025014	card19	stop_lost
6	19,741,585	G	ENSSAUG00010025014	card19	stop_lost
6	20,428,092	A	ENSSAUG00010000831	dstyk	stop_gained
6	20,570,980	A	ENSSAUG00010002489	-	stop_gained

6	20,785,353	T	ENSSAUG00010003779	thumpd3	stop_gained
6	21,388,170	A	ENSSAUG00010013342	-	stop_gained
6	21,699,210	T	ENSSAUG00010014159	prkcda	splice_donor_variant
6	21,699,210	T	ENSSAUG00010014159	prkcda	splice_donor_variant
6	21,699,210	T	ENSSAUG00010014159	prkcda	splice_donor_variant
6	21,810,238	T	ENSSAUG00010015226	erbb3a	stop_gained
6	22,064,354	T	ENSSAUG00010000527	-	stop_gained
6	22,091,471	T	ENSSAUG00010000544	-	stop_gained
6	22,167,505	G	ENSSAUG00010000558	NEUROD4	stop_lost
6	22,479,353	T	ENSSAUG00010023012	slc4a8	stop_gained
6	22,687,968	A	ENSSAUG00010023977	esyt1b	splice_donor_variant
6	22,691,890	T	ENSSAUG00010023977	esyt1b	splice_donor_variant
6	22,693,336	G	ENSSAUG00010023977	esyt1b	splice_acceptor_variant
6	22,700,591	A	ENSSAUG00010023977	esyt1b	stop_gained
6	22,702,061	G	ENSSAUG00010023977	esyt1b	stop_gained
6	22,702,111	G	ENSSAUG00010023977	esyt1b	stop_lost
6	23,056,527	G	ENSSAUG00010024301	pip4k2cb	start_lost
6	23,407,018	T	ENSSAUG00010025152	ANKRD52	stop_gained
6	27,184,744	T	ENSSAUG00010003911	pcsk1	stop_gained,splice_region_variant
6	27,627,994	G	ENSSAUG00010006718	Mx1	splice_acceptor_variant
6	27,800,454	C	ENSSAUG00010007036	adamts9	stop_gained
6	27,800,454	C	ENSSAUG00010007036	adamts9	stop_gained
6	27,800,454	C	ENSSAUG00010007036	adamts9	stop_gained
6	28,266,426	T	ENSSAUG00010007915	-	stop_gained
6	28,323,358	T	ENSSAUG00010007929	suclg2	stop_gained
6	28,323,358	T	ENSSAUG00010007929	suclg2	stop_gained
6	28,394,700	C	ENSSAUG00010007929	suclg2	splice_donor_variant
6	29,015,976	A	ENSSAUG00010010704	hmces	stop_gained
6	29,026,131	A	ENSSAUG00010010721	uqcrc1	splice_donor_variant
6	29,142,811	T	ENSSAUG00010000378	si:dkey-202e22.2	stop_gained
6	29,172,133	C	ENSSAUG00010000485	dnah1	splice_donor_variant
6	29,197,382	T	ENSSAUG00010000892	mapkapk3	splice_donor_variant
6	29,262,645	A	ENSSAUG00010000992	rae1	splice_donor_variant
6	29,277,130	A	ENSSAUG00010001062	bmp7a	splice_donor_variant
6	29,280,816	T	ENSSAUG00010001064	-	stop_gained
6	29,432,203	A	ENSSAUG00010001139	cdh4	stop_gained
6	29,720,416	A	ENSSAUG00010006601	ogfrl2	splice_acceptor_variant
6	29,882,992	T	ENSSAUG00010010207	sulf2a	splice_donor_variant
6	29,976,684	A	ENSSAUG00010011005	ndrg3a	stop_gained
6	29,988,008	A	ENSSAUG00010012056	trpc4apa	stop_gained
6	29,992,479	A	ENSSAUG00010012056	trpc4apa	splice_donor_variant
6	29,992,479	A	ENSSAUG00010012056	trpc4apa	splice_donor_variant
6	29,992,479	A	ENSSAUG00010012056	trpc4apa	splice_donor_variant
6	29,992,479	A	ENSSAUG00010012056	trpc4apa	splice_donor_variant

6	29,994,673	C	ENSSAUG00010012056	trpc4apa	splice_acceptor_variant
6	30,002,744	T	ENSSAUG00010013596	myh7ba	splice_donor_variant
6	30,002,847	C	ENSSAUG00010013596	myh7ba	splice_acceptor_variant
6	30,007,560	G	ENSSAUG00010013596	myh7ba	splice_donor_variant
6	30,007,565	T	ENSSAUG00010013596	myh7ba	stop_gained
6	30,009,927	T	ENSSAUG00010013596	myh7ba	splice_donor_variant
6	30,049,463	G	ENSSAUG00010013623	-	start_lost
6	30,157,474	T	ENSSAUG00010014373	hcfc1a	splice_acceptor_variant
6	30,157,474	T	ENSSAUG00010014373	hcfc1a	splice_acceptor_variant
6	30,174,362	A	ENSSAUG00010014432	opn1sw2	splice_donor_variant
6	30,174,586	A	ENSSAUG00010014432	opn1sw2	splice_acceptor_variant
6	30,215,933	T	ENSSAUG00010014492	cxxc1b	stop_gained
6	30,259,105	T	ENSSAUG00010014567	SRPK3	splice_acceptor_variant
6	30,259,105	T	ENSSAUG00010014567	SRPK3	splice_acceptor_variant
6	30,259,105	T	ENSSAUG00010014567	SRPK3	splice_acceptor_variant
6	30,259,105	T	ENSSAUG00010014567	SRPK3	splice_acceptor_variant
6	30,259,105	T	ENSSAUG00010014567	SRPK3	splice_acceptor_variant
6	30,274,696	T	ENSSAUG00010017188	grm6b	stop_gained
6	34,533,046	G	ENSSAUG00010018076	fbln2	stop_gained
6	34,555,978	A	ENSSAUG00010018076	fbln2	splice_donor_variant
6	34,976,226	C	ENSSAUG00010018186	fam120a	splice_donor_variant
6	35,011,678	T	ENSSAUG00010018190	cass4	stop_gained
6	35,011,678	T	ENSSAUG00010018190	cass4	stop_gained
6	37,429,304	T	ENSSAUG00010001684	plekha6	splice_donor_variant
6	37,429,304	T	ENSSAUG00010001684	plekha6	splice_donor_variant
6	37,429,304	T	ENSSAUG00010001684	plekha6	splice_donor_variant
6	37,429,304	T	ENSSAUG00010001684	plekha6	splice_donor_variant
6	37,429,304	T	ENSSAUG00010001684	plekha6	splice_donor_variant
6	37,537,169	C	ENSSAUG00010002761	ALDH1L1	stop_lost
6	37,537,169	C	ENSSAUG00010002761	ALDH1L1	stop_lost
9	16,167,058	A	ENSSAUG00010014986	-	splice_donor_variant
9	16,170,741	G	ENSSAUG00010014986	-	stop_lost
9	16,400,789	A	ENSSAUG00010015009	dop1b	splice_donor_variant
9	16,400,789	A	ENSSAUG00010015009	dop1b	splice_donor_variant
9	16,459,929	A	ENSSAUG00010015074	casq2	stop_gained
9	16,728,696	T	ENSSAUG00010015320	-	stop_gained
9	16,728,696	T	ENSSAUG00010015320	-	stop_gained
9	16,728,696	T	ENSSAUG00010015320	-	stop_gained
9	16,728,696	T	ENSSAUG00010015320	-	stop_gained
9	16,728,696	T	ENSSAUG00010015320	-	stop_gained
9	16,728,696	T	ENSSAUG00010015320	-	stop_gained
9	16,728,696	T	ENSSAUG00010015320	-	stop_gained
9	16,728,696	T	ENSSAUG00010015320	-	stop_gained
9	16,728,696	T	ENSSAUG00010015320	-	stop_gained
9	16,728,696	T	ENSSAUG00010015320	-	stop_gained
9	16,729,367	T	ENSSAUG00010015320	-	stop_gained

9	16,729,367	T	ENSSAUG00010015320	-	stop_gained
9	16,729,367	T	ENSSAUG00010015320	-	stop_gained
9	18,060,493	A	ENSSAUG00010021366	stk24a	stop_gained
9	18,060,493	A	ENSSAUG00010021366	stk24a	stop_gained
9	21,512,679	T	ENSSAUG00010027408	xrcc5	splice_donor_variant
9	21,512,680	A	ENSSAUG00010027408	xrcc5	splice_donor_variant
9	21,757,990	C	ENSSAUG00010027611	-	stop_gained
9	21,759,656	C	ENSSAUG00010027611	-	start_lost
9	22,195,730	T	ENSSAUG00010027671	ptprna	stop_gained
11	20,619,984	T	ENSSAUG00010027717	ptprsa	stop_gained
11	20,620,351	T	ENSSAUG00010027717	ptprsa	start_lost
11	21,277,161	T	ENSSAUG00010005991	uhrf1	start_lost
11	21,347,395	T	ENSSAUG00010006107	jak3	stop_gained
11	21,350,423	C	ENSSAUG00010007447	-	splice_donor_variant
11	21,483,392	G	ENSSAUG00010007484	mast3b	stop_lost
11	21,483,422	A	ENSSAUG00010007484	mast3b	stop_gained
11	21,484,789	A	ENSSAUG00010007484	mast3b	splice_donor_variant
11	21,484,789	A	ENSSAUG00010007484	mast3b	splice_donor_variant
11	21,484,789	A	ENSSAUG00010007484	mast3b	splice_donor_variant
17	4,558,556	C	ENSSAUG00010003783	cnot10	splice_donor_variant
17	4,558,556	C	ENSSAUG00010003783	cnot10	splice_donor_variant
17	4,558,556	C	ENSSAUG00010003783	cnot10	splice_donor_variant
17	4,797,971	A	ENSSAUG00010007245	-	stop_gained
17	5,121,820	A	ENSSAUG00010007302	-	stop_gained
17	5,121,820	A	ENSSAUG00010007302	-	stop_gained
17	18,885,092	T	ENSSAUG00010021226	-	splice_donor_variant
17	18,926,927	T	ENSSAUG00010021238	-	splice_donor_variant
18	15,594,896	T	ENSSAUG00010017998	il1rapl2	splice_acceptor_variant
18	15,594,896	T	ENSSAUG00010017998	il1rapl2	splice_acceptor_variant
18	31,554,538	A	ENSSAUG00010015403	med12	stop_lost
18	31,554,674	C	ENSSAUG00010015403	med12	splice_acceptor_variant
18	31,554,705	T	ENSSAUG00010015403	med12	splice_donor_variant
18	31,555,189	C	ENSSAUG00010015403	med12	stop_lost
18	31,556,781	T	ENSSAUG00010015403	med12	stop_gained
18	31,556,781	T	ENSSAUG00010015403	med12	stop_gained
18	31,567,639	C	ENSSAUG00010015403	med12	stop_gained
18	31,567,639	C	ENSSAUG00010015403	med12	stop_gained
18	31,585,170	G	ENSSAUG00010015424	-	splice_donor_variant
18	31,585,170	G	ENSSAUG00010015424	-	splice_donor_variant
18	31,598,519	T	ENSSAUG00010015424	-	stop_gained
18	31,601,430	T	ENSSAUG00010015424	-	splice_donor_variant
18	31,957,979	G	ENSSAUG00010015745	-	start_lost
18	32,090,408	G	ENSSAUG00010015763	SLC4A11	start_lost
18	32,090,798	T	ENSSAUG00010015763	SLC4A11	splice_acceptor_variant
18	32,090,832	A	ENSSAUG00010015763	SLC4A11	stop_gained
18	32,218,796	T	ENSSAUG00010016267	smyd5	splice_acceptor_variant

18	32,218,796	T	ENSSAUG00010016267	smyd5	splice_acceptor_variant
18	32,219,210	A	ENSSAUG00010016267	smyd5	stop_gained
18	32,219,403	A	ENSSAUG00010016267	smyd5	start_lost
18	32,228,654	G	ENSSAUG00010017169	sfxn5b	splice_donor_variant
18	32,238,212	T	ENSSAUG00010017175	-	splice_donor_variant
18	32,247,183	G	ENSSAUG00010017177	-	stop_lost
18	32,247,260	A	ENSSAUG00010017177	-	splice_acceptor_variant
18	32,335,270	T	ENSSAUG00010017273	atp8a1	splice_acceptor_variant
18	32,335,270	T	ENSSAUG00010017273	atp8a1	splice_acceptor_variant
18	32,335,320	A	ENSSAUG00010017273	atp8a1	splice_acceptor_variant
18	32,335,320	A	ENSSAUG00010017273	atp8a1	splice_acceptor_variant
18	32,335,320	A	ENSSAUG00010017273	atp8a1	splice_acceptor_variant
18	32,371,403	C	ENSSAUG00010017273	atp8a1	splice_donor_variant
18	32,422,020	T	ENSSAUG00010022108	slc30a9	splice_donor_variant
18	32,696,108	G	ENSSAUG00010022821	RAB28	stop_lost
18	32,793,475	C	ENSSAUG00010022862	gpc2	start_lost
18	32,799,383	C	ENSSAUG00010022862	gpc2	splice_donor_variant
18	32,799,383	C	ENSSAUG00010022862	gpc2	splice_donor_variant
18	32,799,383	C	ENSSAUG00010022862	gpc2	splice_donor_variant
18	32,799,383	C	ENSSAUG00010022862	gpc2	splice_donor_variant
18	32,799,384	T	ENSSAUG00010022862	gpc2	splice_donor_variant
18	32,799,384	T	ENSSAUG00010022862	gpc2	splice_donor_variant
18	32,799,384	T	ENSSAUG00010022862	gpc2	splice_donor_variant
18	32,799,384	T	ENSSAUG00010022862	gpc2	splice_donor_variant
18	32,810,702	T	ENSSAUG00010022943	-	stop_gained
18	32,810,702	T	ENSSAUG00010022943	-	stop_gained
18	32,814,389	A	ENSSAUG00010022943	-	stop_gained
18	32,814,389	A	ENSSAUG00010022943	-	stop_gained
18	32,814,389	A	ENSSAUG00010022943	-	stop_gained
18	32,818,918	A	ENSSAUG00010022943	-	stop_gained
18	32,958,078	A	ENSSAUG00010022971	-	splice_donor_variant
19	2,002,552	T	ENSSAUG00010016425	-	stop_gained
22	13,235,768	C	ENSSAUG00010015670	aven	stop_gained
24	20,104,631	T	ENSSAUG00010026221	-	splice_donor_variant,non_coding_transcript_variant
24	20,357,071	T	ENSSAUG00010026252	-	start_lost

Table S7: Non-redundant Gene Ontology (GO) Term list for the different comparisons, including GO term code, GO term biological process name, fold enrichment, false discovery rate (FDR) value and genes included in the enrichment.

**A-B**				
**Go term**	**GO name**	**fold enrichment**	**fdr**	**genes**
GO:0008152	metabolic process	1.70	0.000	myorg;ube2r2;espl1;ell2;rps26;ruvbl1;cnr1;bpnt1;uroc1;rad18;idh3b;iars1;rngtt;hace1;ptpdc1;prkag1;mettl1;mtif3;gla;traip;cnot10;tarbp2;hadhb;kbtbd8;ubap1;rbm5;nudt14;phf2;tsfm;rwdd1;cxxc1b;edem1;fmr1;ctsz;eogt;rrp9;hdac3;alas1;nudt2;ppp4r1;adamts9;dcaf12;hmces;smug1;rft1;ptprk;mmp19;lyar;rpl29;mst1;cers5;mars2;mars1;eefsec;rpl36a;usp4;os9;crbn;rplp0;btk;camkv;thumpd3;tpgs2;aldh1l1;timeless;pcsk1;gnl3l;cdk4;src;fancd2;cdk2;suclg2;apof;rae1;extl3;h6pd;rpn1;trpc4apa;rars2;celf4;vhl;ptpra;parp3;thoc7;npepl1;bcdin3d;psmd6;suox;mapkapk3
GO:0006139	nucleobase-containing compound metabolic process	2.36	0.002	ell2;smug1;ruvbl1;rad18;lyar;iars1;mars2;rngtt;mars1;mettl1;traip;cnot10;thumpd3;timeless;tarbp2;gnl3l;fancd2;suclg2;rbm5;nudt14;h6pd;cxxc1b;fmr1;rars2;celf4;vhl;parp3;thoc7;rrp9;bcdin3d;nudt2
GO:1901360	organic cyclic compound metabolic process	2.27	0.002	ell2;smug1;ruvbl1;rad18;lyar;iars1;mars2;rngtt;mars1;mettl1;traip;cnot10;thumpd3;aldh1l1;timeless;tarbp2;gnl3l;fancd2;suclg2;apof;rbm5;nudt14;h6pd;cxxc1b;fmr1;rars2;celf4;vhl;parp3;thoc7;rrp9;bcdin3d;alas1;nudt2
GO:0071704	organic substance metabolic process	1.76	0.000	myorg;ube2r2;espl1;ell2;rps26;ruvbl1;cnr1;bpnt1;uroc1;rad18;idh3b;iars1;rngtt;hace1;prkag1;mettl1;mtif3;gla;traip;cnot10;tarbp2;hadhb;kbtbd8;ubap1;rbm5;nudt14;phf2;tsfm;rwdd1;cxxc1b;edem1;fmr1;ctsz;eogt;rrp9;hdac3;alas1;nudt2;ppp4r1;adamts9;dcaf12;hmces;smug1;rft1;ptprk;mmp19;lyar;rpl29;mst1;cers5;mars2;mars1;eefsec;rpl36a;usp4;os9;crbn;rplp0;btk;camkv;thumpd3;tpgs2;aldh1l1;timeless;pcsk1;gnl3l;cdk4;src;fancd2;cdk2;suclg2;apof;rae1;extl3;h6pd;rpn1;trpc4apa;rars2;celf4;vhl;ptpra;parp3;thoc7;npepl1;bcdin3d;psmd6;mapkapk3
GO:0046483	heterocycle metabolic process	2.37	0.001	ell2;smug1;ruvbl1;rad18;lyar;iars1;mars2;rngtt;mars1;mettl1;traip;cnot10;thumpd3;aldh1l1;timeless;tarbp2;gnl3l;fancd2;suclg2;rbm5;nudt14;h6pd;cxxc1b;fmr1;rars2;celf4;vhl;parp3;thoc7;rrp9;bcdin3d;alas1;nudt2
GO:0044237	cellular metabolic process	1.79	0.000	ube2r2;ell2;rps26;ruvbl1;cnr1;bpnt1;uroc1;rad18;idh3b;iars1;rngtt;hace1;ptpdc1;prkag1;mettl1;mtif3;gla;traip;cnot10;tarbp2;hadhb;ubap1;rbm5;nudt14;tsfm;rwdd1;cxxc1b;edem1;fmr1;eogt;rrp9;alas1;nudt2;ppp4r1;smug1;rft1;ptprk;lyar;rpl29;cers5;mars2;mars1;eefsec;rpl36a;usp4;os9;crbn;rplp0;btk;camkv;thumpd3;aldh1l1;timeless;pcsk1;gnl3l;cdk4;src;fancd2;cdk2;suclg2;extl3;h6pd;rpn1;trpc4apa;rars2;celf4;vhl;ptpra;parp3;thoc7;bcdin3d;psmd6;suox;mapkapk3

GO:0009987	cellular process	1.29	0.001	ube2r2;ell2;rab33a;rabif;idh3b;reps1;iars1;rngtt;apcdd1l;fkbp11;oxtr;prkag1;stx18;cass4;stx16;gla;olfml3;hadhb;rab22a;myh7ba;kbtbd8;clu;rwdd1;edem1;fmr1;eogt;otop1;rrp9;hdac3;cplane2;nudt2;sla2;rft1;ptprk;kpna5;srgap3;tespa1;lyar;prph;ndrg3a;timm8a;rpl36a;rplp0;ddit3;brk1;btk;thumpd3;pcsk1;gnl3l;atxn7;cdk4;fancd2;cdk2;mon1a;pfdn5;pdrg1;h6pd;rpn1;cav3;smyd5;id1;cidec;sp7;ptpra;parp3;thoc7;fbln2;myorg;espl1;rps26;ruvbl1;cnr1;bpnt1;uroc1;b9d2;rad18;hace1;ptpdc1;dnah1;mcrs1;mettl1;mtif3;slc4a8;traip;cnot10;tarbp2;ubap1;rbm5;grm6b;nudt14;phf2;tsfm;cxxc1b;dctn2;cse1l;esyt1b;alas1;gli1;slc35a1;ppp4r1;adamts9;hmces;med12;smug1;cdh4;poc1a;aggf1;scg5;mmp19;strip1;nckap1l;rpl29;cers5;mars2;mars1;eefsec;usp4;os9;crbn;col7a1;camkv;knl1;aldh1l1;timeless;src;suclg2;scara3;rae1;extl3;nop56;slc4a11;trpc4apa;stau1;rars2;emc3;celf4;vhl;smarcd1;bcdin3d;psmd6;suox;mapkapk3;il1rapl2
GO:0044238	primary metabolic process	1.82	0.000	myorg;ube2r2;espl1;ell2;rps26;ruvbl1;cnr1;bpnt1;uroc1;rad18;idh3b;iars1;rngtt;hace1;prkag1;mettl1;mtif3;gla;traip;cnot10;tarbp2;hadhb;kbtbd8;ubap1;rbm5;nudt14;phf2;tsfm;rwdd1;cxxc1b;edem1;fmr1;ctsz;eogt;rrp9;hdac3;alas1;nudt2;ppp4r1;adamts9;dcaf12;hmces;smug1;rft1;ptprk;mmp19;lyar;rpl29;mst1;cers5;mars2;mars1;eefsec;rpl36a;usp4;os9;crbn;rplp0;btk;camkv;thumpd3;tpgs2;timeless;pcsk1;gnl3l;cdk4;src;fancd2;cdk2;suclg2;apof;extl3;h6pd;rpn1;trpc4apa;rars2;celf4;vhl;ptpra;parp3;thoc7;npepl1;bcdin3d;psmd6;mapkapk3
GO:0006725	cellular aromatic compound metabolic process	2.35	0.001	ell2;smug1;ruvbl1;rad18;lyar;iars1;mars2;rngtt;mars1;mettl1;traip;cnot10;thumpd3;aldh1l1;timeless;tarbp2;gnl3l;fancd2;suclg2;rbm5;nudt14;h6pd;cxxc1b;fmr1;rars2;celf4;vhl;parp3;thoc7;rrp9;bcdin3d;alas1;nudt2
GO:0034641	cellular nitrogen compound metabolic process	2.57	0.000	ell2;rps26;smug1;ruvbl1;uroc1;rad18;lyar;iars1;rpl29;cers5;mars2;rngtt;mars1;eefsec;rpl36a;rplp0;mettl1;mtif3;gla;traip;cnot10;thumpd3;aldh1l1;timeless;pcsk1;tarbp2;gnl3l;fancd2;suclg2;rbm5;nudt14;tsfm;rwdd1;h6pd;cxxc1b;fmr1;rars2;celf4;vhl;parp3;thoc7;rrp9;bcdin3d;alas1;nudt2
GO:0006807	nitrogen compound metabolic process	1.89	0.000	ube2r2;espl1;ell2;rps26;ruvbl1;cnr1;uroc1;rad18;iars1;rngtt;hace1;prkag1;mettl1;mtif3;gla;traip;cnot10;tarbp2;kbtbd8;ubap1;rbm5;nudt14;phf2;tsfm;rwdd1;cxxc1b;edem1;fmr1;ctsz;eogt;rrp9;hdac3;alas1;nudt2;ppp4r1;adamts9;dcaf12;hmces;smug1;rft1;ptprk;mmp19;lyar;rpl29;mst1;cers5;mars2;mars1;eefsec;rpl36a;usp4;os9;crbn;rplp0;btk;camkv;thumpd3;tpgs2;aldh1l1;timeless;pcsk1;gnl3l;cdk4;src;fancd2;cdk2;suclg2;extl3;h6pd;rpn1;trpc4apa;rars2;celf4;vhl;ptpra;parp3;thoc7;npepl1;bcdin3d;psmd6;mapkapk3
GO:1901564	organonitrogen compound metabolic process	1.89	0.000	ube2r2;espl1;rps26;ruvbl1;cnr1;uroc1;rad18;iars1;hace1;prkag1;mtif3;gla;traip;kbtbd8;ubap1;phf2;tsfm;rwdd1;cxxc1b;edem1;ctsz;eogt;hdac3;alas1;nudt2;ppp4r1;adamts9;dcaf12;hmces;rft1;ptprk;mmp19;rpl29;mst1;cers5;mars2;mars1;eefsec;rpl36a;usp4;os9;crbn;rplp0;btk;camkv;tpgs2;aldh1l1;pcsk1;cdk4;src;cdk2;suclg2;extl3;h6pd;rpn1;trpc4apa;rars2;vhl;ptpra;parp3;npepl1;psmd6;mapkapk3
GO:0043603	cellular amide metabolic process	4.16	0.000	rps26;uroc1;suclg2;iars1;rpl29;tsfm;cers5;mars2;mars1;rwdd1;eefsec;rpl36a;rplp0;rars2;mtif3;gla;pcsk1
GO:0006099	tricarboxylic acid cycle	16.40	0.048	suclg2;idh3b
GO:0006412	translation	5.11	0.003	tsfm;mars2;mars1;rwdd1;rps26;eefsec;rpl36a;rplp0;rars2;mtif3;iars1;rpl29

GO:0010467	gene expression	2.48	0.008	ell2;rps26;lyar;iars1;rpl29;mars2;rngtt;mars1;eefsec;rpl36a;rplp0;mettl1;mtif3;thumpd3;pcsk1;tarbp2;gnl3l;rbm5;rae1;tsfm;rwdd1;fmr1;rars2;celf4;thoc7;rrp9
GO:0043170	macromolecule metabolic process	1.90	0.000	ube2r2;espl1;ell2;rps26;ruvbl1;rad18;iars1;rngtt;hace1;prkag1;mettl1;mtif3;traip;cnot10;tarbp2;kbtbd8;ubap1;rbm5;phf2;tsfm;rwdd1;cxxc1b;edem1;fmr1;ctsz;eogt;rrp9;hdac3;alas1;ppp4r1;adamts9;dcaf12;hmces;smug1;rft1;ptprk;mmp19;lyar;rpl29;mst1;mars2;mars1;eefsec;rpl36a;usp4;os9;crbn;rplp0;btk;camkv;thumpd3;tpgs2;timeless;pcsk1;gnl3l;cdk4;src;fancd2;cdk2;rae1;extl3;rpn1;trpc4apa;rars2;celf4;vhl;ptpra;parp3;thoc7;npepl1;bcdin3d;psmd6;mapkapk3
GO:0034645	cellular macromolecule biosynthetic process	3.49	0.003	rps26;rft1;iars1;rpl29;extl3;tsfm;mars2;mars1;rwdd1;eefsec;rpl36a;rpn1;rplp0;rars2;eogt;mtif3;alas1
GO:0044260	cellular macromolecule metabolic process	2.76	0.000	ube2r2;rps26;smug1;rft1;ruvbl1;rad18;iars1;rpl29;mars2;mars1;hace1;eefsec;rpl36a;usp4;os9;crbn;rplp0;mettl1;mtif3;traip;cnot10;thumpd3;timeless;fancd2;ubap1;extl3;tsfm;rwdd1;cxxc1b;edem1;rpn1;trpc4apa;rars2;eogt;vhl;parp3;bcdin3d;psmd6;alas1
GO:0009059	macromolecule biosynthetic process	2.75	0.027	ell2;rps26;rft1;iars1;rpl29;extl3;tsfm;mars2;mars1;rwdd1;eefsec;rpl36a;rpn1;rplp0;rars2;eogt;mtif3;alas1
GO:0019538	protein metabolic process	1.96	0.000	ube2r2;espl1;rps26;ruvbl1;rad18;iars1;hace1;prkag1;mtif3;traip;kbtbd8;ubap1;phf2;tsfm;rwdd1;cxxc1b;edem1;ctsz;eogt;hdac3;alas1;ppp4r1;adamts9;dcaf12;hmces;rft1;ptprk;mmp19;rpl29;mst1;mars2;mars1;eefsec;rpl36a;usp4;os9;crbn;rplp0;btk;camkv;tpgs2;pcsk1;cdk4;src;cdk2;extl3;rpn1;trpc4apa;rars2;vhl;ptpra;parp3;npepl1;psmd6;mapkapk3
GO:0034660	ncRNA metabolic process	3.98	0.024	mars2;tarbp2;gnl3l;mars1;ell2;rars2;mettl1;rrp9;lyar;bcdin3d;thumpd3;iars1
GO:0090304	nucleic acid metabolic process	2.48	0.006	ell2;smug1;ruvbl1;rad18;lyar;iars1;mars2;rngtt;mars1;mettl1;traip;cnot10;thumpd3;timeless;tarbp2;gnl3l;fancd2;rbm5;cxxc1b;fmr1;rars2;celf4;vhl;parp3;thoc7;rrp9;bcdin3d
GO:0048513	animal organ development	1.93	0.025	adamts9;myorg;med12;cdh4;ruvbl1;cnr1;irf6;aggf1;mst1;hace1;apcdd1l;oxtr;mcrs1;crbn;stx16;brk1;barx1;aldh1l1;atxn7;src;kbtbd8;extl3;nop56;cxxc1b;cav3;fmr1;dctn2;rars2;emc3;smyd5;otop1;vhl;sp7;ptpra;smarcd1;psmd6;hdac3;alas1;gli1
**A-C**				
**Go term**	**GO name**	**fold enrichment**	**fdr**	
GO:0001894	tissue homeostasis	17.26	0.046	tert; nkiras1;

GO:0090304	nucleic acid metabolic process	3.08	0.047	ercc1;tert;mcm2;uhrf1;xrcc5;txnl4a;srfbp1;nsun2;trit1;sf3a3;tent4a;ccdc124;top2b;nr1i2;trim71;cpsf1;smg9;yars1
GO:0034641	cellular nitrogen compound metabolic process	2.75	0.009	rps27a;ercc1;tert;gsr;mcm2;xrcc5;st3gal1;nsun2;tent4a;ccdc124;top2b;nr1i2;trim71;cpsf1;eif3i;mrps18b;yars1;rpe;rpl15;uhrf1;rpl14;txnl4a;srfbp1;trit1;sf3a3;psenen;smg9
GO:0044260	cellular macromolecule metabolic process	2.83	0.037	rps27a; ercc1;rbbp5;tert;has1;mcm2;rpl15;uhrf1;rpl14;xrcc5;st3gal1;nsun2;pigu;top2b;psmf1;trim71;ngly1;prmt2;eif3i;smg9;mrps18b;yars1
**B-C**				
**Go term**	**GO name**	**fold enrichment**	**fdr**	**genes**
GO:0070920	regulation of production of small RNA involved in gene silencing by RNA	101.46	0.018	tarbp2;prkra;bcdin3d
GO:0048705	skeletal system morphogenesis	6.41	0.011	bcl9;med12;irf6;sp7;hoxd9;hoxd4;hoxd3;nkx3-2;aldh1l1;chrna1
GO:0001501	skeletal system development	4.34	0.022	bcl9;med12;src;fmr1;irf6;sp7;hoxd9;hoxd4;hoxd3;nkx3-2;aldh1l1;chrna1
GO:0032502	developmental process	1.71	0.017	myorg;rab33a;ruvbl1;evx2;mfap2;chn1;b9d2;tspo;agr2;hoxd9;hoxd4;hoxd3;nkx3-2;apcdd1l;top2b;oxtr;aplp1;celsr2;dstyk;nudc;fmr1;dctn2;hdac3;alas1;gli1;med12;nphs1;vwa1;irf6;nbl1;aggf1;col22a1;chaf1b;nckap1l;chrna1;rpl36a;usp4;nr1i2;crbn;rplp0;brk1;btk;capzb;bzw2;ilf2;hoxd10;aldh1l1;hoxd11;bcl9;src;bap1;cav3;hspb6;stau1;emc3;smyd5;vhl;sp7;parp3;smarcd1;sp9
GO:0048513	animal organ development	2.13	0.008	myorg;med12;ruvbl1;evx2;mfap2;nphs1;vwa1;irf6;tspo;nbl1;aggf1;hoxd9;col22a1;hoxd4;hoxd3;chaf1b;nkx3-2;chrna1;apcdd1l;top2b;oxtr;crbn;brk1;celsr2;capzb;ilf2;aldh1l1;dstyk;bcl9;src;nudc;cav3;fmr1;hspb6;dctn2;emc3;smyd5;vhl;sp7;smarcd1;hdac3;alas1;gli1
GO:0009790	embryo development	3.01	0.003	med12;mfap2;irf6;tspo;nbl1;aggf1;hoxd9;col22a1;hoxd4;hoxd3;chaf1b;nkx3-2;rpl36a;crbn;rplp0;celsr2;aldh1l1;dstyk;bcl9;cav3;fmr1;dctn2;smyd5;smarcd1;sp9;gli1
GO:0048568	embryonic organ development	3.69	0.013	dstyk;bcl9;med12;mfap2;irf6;tspo;nbl1;aggf1;hoxd9;hoxd4;hoxd3;chaf1b;nkx3-2;crbn;cav3;smyd5;aldh1l1
GO:0006412	translation	4.68	0.023	mars2;mars1;rps26;eefsec;mrpl20;rpl36a;rplp0;eif4enif1;mrps18b;iars1;rpl29

GO:0043603	cellular amide metabolic process	3.50	0.020	rps26;mrpl20;uroc1;gsr;eif4enif1;iars1;rpl29;cers5;mars2;mars1;eefsec;rpl36a;rplp0;psenen;gla;mrps18b
GO:0034641	cellular nitrogen compound metabolic process	2.30	0.004	rps26;ruvbl1;uroc1;prkra;gsr;rad18;hoxd9;iars1;rpl29;cers5;mars2;mars1;eefsec;top2b;ccdc36;rpl36a;nr1i2;rplp0;hnrnpa3;gla;thumpd3;mrps18b;aldh1l1;bysl;trub2;tarbp2;mrpl20;fancd2;eif4enif1;rbm5;h6pd;mcm7;fmr1;psenen;smg9;ssu72;celf4;vhl;parp3;rrp9;bcdin3d;alas1
GO:0010467	gene expression	2.39	0.035	rps26;prkra;hoxd9;iars1;rpl29;mars2;mars1;eefsec;rpl36a;nr1i2;rplp0;hnrnpa3;thumpd3;mrps18b;bysl;trub2;tarbp2;mrpl20;zc3h11a;eif4enif1;rbm5;fmr1;ssu72;celf4;rrp9
GO:0044260	cellular macromolecule metabolic process	2.21	0.020	rps26;rft1;ruvbl1;rad18;iars1;rpl29;mars2;mars1;eefsec;top2b;ccdc36;rpl36a;usp4;os9;crbn;rplp0;ngly1;thumpd3;mrps18b;mrpl20;fancd2;has1;eif4enif1;bap1;usp49;mcm7;edem1;smg9;vhl;parp3;bcdin3d;alas1
GO:0090304	nucleic acid metabolic process	2.39	0.027	ruvbl1;prkra;rad18;hoxd9;iars1;mars2;mars1;top2b;ccdc36;nr1i2;hnrnpa3;thumpd3;bysl;trub2;tarbp2;fancd2;rbm5;mcm7;fmr1;smg9;ssu72;celf4;vhl;parp3;rrp9;bcdin3d
